# Geosphere-Biosphere Interactions in *Bio-Activity* Volcanic Lakes: Evidences from Hule and Rìo Cuarto (Costa Rica)

**DOI:** 10.1371/journal.pone.0102456

**Published:** 2014-07-24

**Authors:** Jacopo Cabassi, Franco Tassi, Francesca Mapelli, Sara Borin, Sergio Calabrese, Dmitri Rouwet, Giovanni Chiodini, Ramona Marasco, Bessem Chouaia, Rosario Avino, Orlando Vaselli, Giovannella Pecoraino, Francesco Capecchiacci, Gabriele Bicocchi, Stefano Caliro, Carlos Ramirez, Raul Mora-Amador

**Affiliations:** 1 Dipartimento di Scienze della Terra, University of Florence, Florence, Italy; 2 CNR – Istituto di Geoscienze e Georisorse, Florence, Italy; 3 Department of Food, Environmental and Nutritional Sciences, University of Milan, Milan, Italy; 4 Dipartimento di Scienze della Terra e del Mare, University of Palermo, Palermo, Italy; 5 Istituto Nazionale di Geofisica e Vulcanologia, Sezione di Bologna, Bologna, Italy; 6 Istituto Nazionale di Geofisica e Vulcanologia, Osservatorio Vesuviano, Naples, Italy; 7 Istituto Nazionale di Geofisica e Vulcanologia, Sezione di Palermo, Palermo, Italy; 8 Centro de Investigaciones en Ciencias Geológicas, Escuela Centroamericana de Geología, Red Sismológica Nacional, Universidad de Costa Rica, San Jose, Costa Rica; Oak Ridge National Laboratory, United States of America

## Abstract

Hule and Río Cuarto are maar lakes located 11 and 18 km N of Poás volcano along a 27 km long fracture zone, in the Central Volcanic Range of Costa Rica. Both lakes are characterized by a stable thermic and chemical stratification and recently they were affected by fish killing events likely related to the uprising of deep anoxic waters to the surface caused by rollover phenomena. The vertical profiles of temperature, pH, redox potential, chemical and isotopic compositions of water and dissolved gases, as well as prokaryotic diversity estimated by DNA fingerprinting and massive 16S rRNA pyrosequencing along the water column of the two lakes, have highlighted that different bio-geochemical processes occur in these meromictic lakes. Although the two lakes host different bacterial and archaeal phylogenetic groups, water and gas chemistry in both lakes is controlled by the same prokaryotic functions, especially regarding the CO_2_-CH_4_ cycle. Addition of hydrothermal CO_2_ through the bottom of the lakes plays a fundamental priming role in developing a stable water stratification and fuelling anoxic bacterial and archaeal populations. Methanogens and methane oxidizers as well as autotrophic and heterotrophic aerobic bacteria responsible of organic carbon recycling resulted to be stratified with depth and strictly related to the chemical-physical conditions and availability of free oxygen, affecting both the CO_2_ and CH_4_ chemical concentrations and their isotopic compositions along the water column. Hule and Río Cuarto lakes were demonstrated to contain a CO_2_ (CH_4_, N_2_)-rich gas reservoir mainly controlled by the interactions occurring between geosphere and biosphere. Thus, we introduced the term of *bio-activity* volcanic lakes to distinguish these lakes, which have analogues worldwide (e.g. Kivu: D.R.C.-Rwanda; Albano, Monticchio and Averno: Italy; Pavin: France) from volcanic lakes only characterized by geogenic CO_2_ reservoir such as Nyos and Monoun (Cameroon).

## Introduction

Volcanic lakes are peculiar natural systems on Earth, although they are a common feature of volcanic systems characterized by recent activity, being present in 476 volcanic structures worldwide (VHub, CVL Group page; [1]). A volcanic lake simultaneously acts as both a calorimeter and a condenser for acidic volatiles from magmatic and hydrothermal degassing [2]–[6]. Thus, its existence and durability strictly depends on the balance between i) inputs of meteoric water and hydrothermal-magmatic fluids and ii) losses related to evaporation, permeation through sediments and streaming [7]. Volcanic lakes were basically classified, as follows [1], [4]: i) “high-activity” lakes affected by the addition of significant amounts of hot and hyperacidic hydrothermal–magmatic fluids; ii) “low-activity” lakes, characterized by CO_2_-dominated fluid inputs at a relatively low rate from sub-lacustrine fluids discharges, favoring the establishment of a stable vertical stratification and possibly the accumulation of high amounts of dissolved gases in the deep water layers. At these conditions, a lake overturn triggered by either i) external events, such as earthquakes, landslides or extreme weather conditions or ii) the progressive attainment of gas saturation conditions may cause the abrupt release of toxic gas clouds in the atmosphere. This phenomenon, also known as “limnic eruption”, was firstly documented at Monoun and Nyos lakes (Cameroon) in 1984 and 1986, respectively [8]–[15]. Accordingly, low activity lakes are commonly indicated as “Nyos-type” lakes.

**Figure 1:**
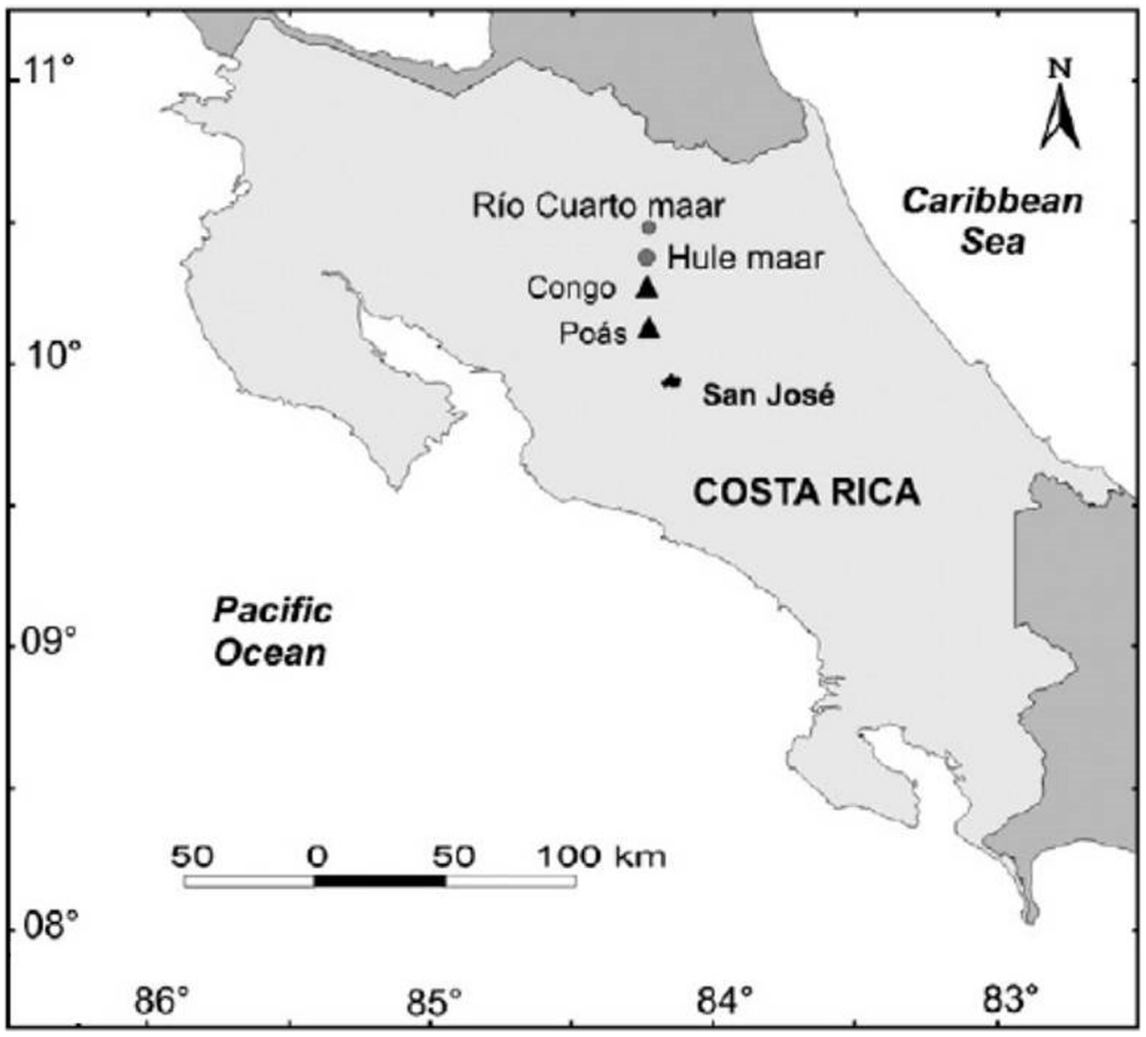
Map of Costa Rica with the location of Hule and Río Cuarto lakes. Modified after Alvarado *et al.* [18].

In Costa Rica, volcanic lakes are found in quiescent systems (Congo and Barva), as well as in volcanoes characterized by moderate hydrothermal activity (Irazú and Tenorio) and strong magmatic fluid emissions (Rincón de la Vieja and Poás) [16], [17]. Hule and Río Cuarto are low-activity, Nyos-type, maar lakes located at 11 and 18 km N of Poás volcano (Fig. 1), respectively, in relation of a 27 km long fracture zone passing through the Sabana Redonda cinder cones, the Poás summit craters (Botos, Active Crater and Von Frantzius) and the Congo stratocone [18]. In these two lakes, changes in the water color and fish death events were repeatedly reported, suggesting the occurrence of rollover episodes related to inputs of deep-originated gases [18]. To the best of our knowledge, no information is available on these lakes for chemical and isotopic compositions of dissolved gases deriving from geogenic sources and the structure of prokaryotic communities. The latter are expected to play pivotal ecological functions, encompassing nutrient remineralization and carbon cycling, which is firmly linked to the fate of dissolved C_1_ gases, i.e. CH_4_ and CO_2_.

This paper presents the geochemical (water and dissolved gas chemistry) and microbiological results obtained from samples collected in 2010 during the 7^th^ Workshop of the Commission on Volcanic Lakes (CVL; Costa Rica 10–21 March 2010), which is part of the International Association of Volcanology and Chemistry of the Earth's Interior (IAVCEI), by a group of geochemists, limnologists, biologists and volcanologists from different universities and scientific institutions. The aim of this multidisciplinary research was to unravel the bio-geochemical processes controlling the physical-chemical features of Hule and Río Cuarto lakes along the vertical profiles, showing their implications for lake stratification and stability, and proposing evidences for a new classification system.

## Morphological and Limnological Outlines

### 2.1 Morphological features

**Figure 2:**
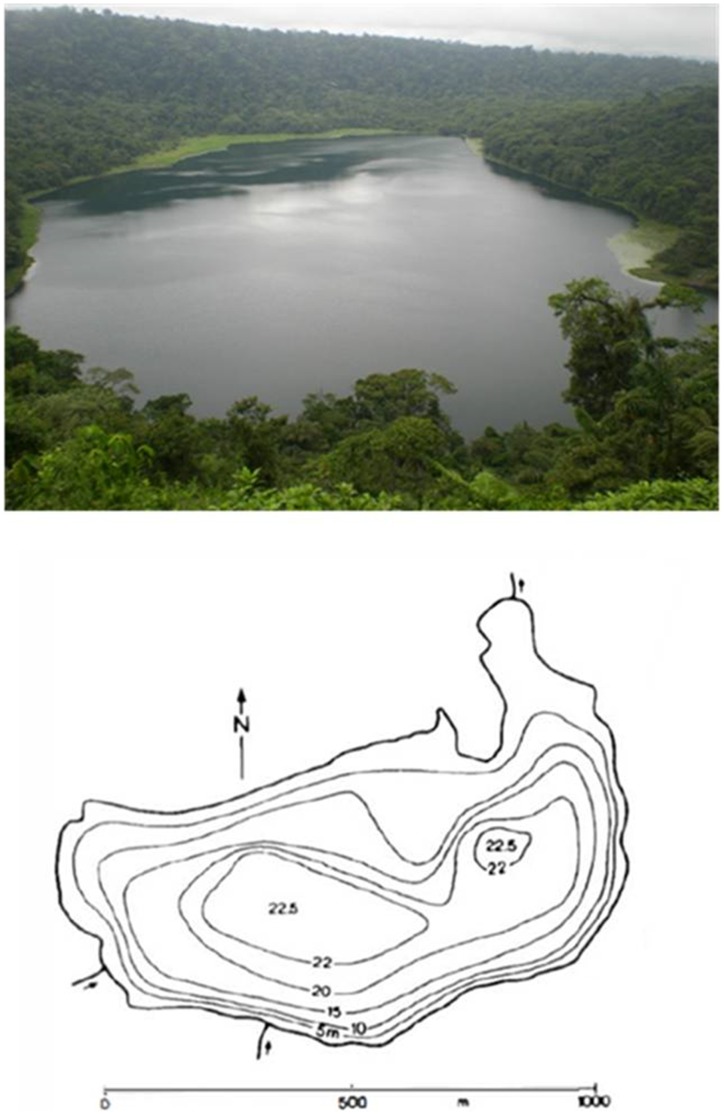
Panoramic view and bathymetric map of Lake Hule (modified after Göcke [24]).

Lake Hule (10°17′42″N, 84°12′37″W) lies within the 2.3×1.8 km wide Hule basin, a volcanic depression also hosting Lake Congo to the north, which is separated from Lake Hule by a volcanic cone, and Lake Bosque Alegre (unofficial name) [18]–[20]. Lake Hule has a half-moon shape, a surface area of about 5.5×10^5^ m^2^, an estimated water volume of 6.9×10^6^ m^3^, and a maximum depth of ∼23 m [17], [18], [21], [22] (Fig. 2). The northern shoreline of the lake shows three tributaries, whereas an emissary (Río Hule) is located to the NE [18], [23], [24].

**Figure 3:**
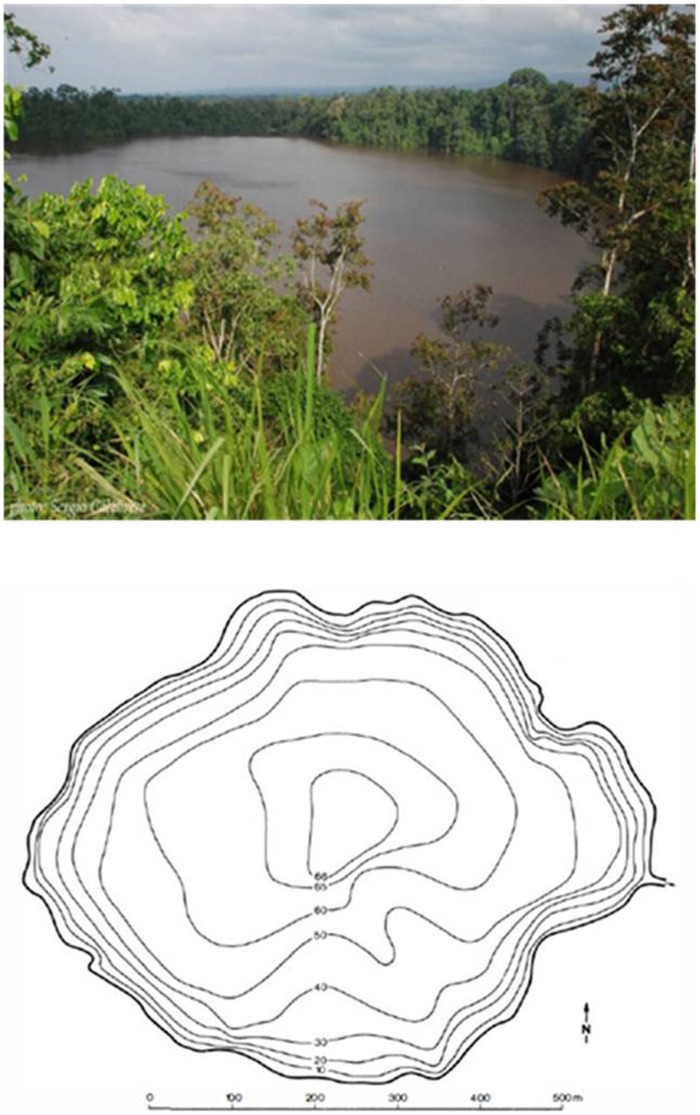
Panoramic view and bathymetric map of Lake Río Cuarto (modified after Göcke *et al.* [25]).

Río Cuarto maar (10°21′23″N, 84°13′00″W) has a rim whose maximum elevation is ∼52 m a.s.l. Lake Río Cuarto shows steep sided walls and a flat bottom, a morphology typical of maar lakes. The lake has an E-W axis of 758 m, a mean width of 581 m, a surface of 3.3×10^5^ m^2^ and a water volume of 15×10^6^ m^3^ [18], [25] (Fig. 3). Río Cuarto is the deepest (∼67 m) natural lake in Costa Rica [19]. A small tributary is located on the eastern shore, whereas no emissaries were recognized [25].

The main morphological features of Hule and Río Cuarto lakes can be summarized using the “depth-ratio” [26], which is a dimensionless parameter equal to the ratio between the average depth (the volume divided by the surface area of the lake) and the maximum depth of the lake. The obtained results are 0.55 and 0.68, respectively, for Lake Hule and Lake Río Cuarto, corresponding to an average depth of 12.6 and 45.5 m. According to Carpenter's heuristic classification [26], the depth-ratio values are consistent with the so-called ellipsoid shape (typical values comprised between 0.5 and 0.66), considered a common feature for volcanic lake basins, even though Río Cuarto morphometry tends to approximate a steep-sided frustum model, corresponding to steep sides and flat bottom [27]. Such morphological features tend to prevent water vertical mixing, favoring meromictic conditions [28]. Thus, these physical parameters have a strong influence on the vertical distribution of chemical species, especially approaching the lake bottom where bio-geochemical processes have their maximum efficiency [29].

### 2.2 Limnological features and rollover events

At Lake Hule, the limit between epi- and hypolimnion, marked by a very weak thermocline and the complete depletion of O_2_, was reported to occur at a depth ranging between −10 and −12 m [23], [24]. As reported by [22], this lake shows a persistent vertical stratification and the presence of CO_2_ in the deepest water strata. Occurrence of fish death episodes, associated with sudden changes of water color from dark blue to red and strong smell in the lake surroundings, were reported by the local population in the last years (4 to 5 events from 1989 to 2002). These events, which took place during the cool, rainy and windy season (i.e. from December to February), were interpreted as caused by rollover phenomena [16], [17], [18], [30].

The transition between epilimnion and hypolimnion in the meromictic Lake Río Cuarto was measured at 20 and 25 m depth in May-June and January-February, respectively [18], [25]. Rollover events, testified by fish killing and color changes of lake water from green to yellow-reddish, were observed in 1920 [31], between 1978 and 1991 [22], in January 1997 [16] and in February 2010 [18], just one month before our sampling. These events were possibly triggered by cooling of the shallow water layer caused by an anomalous weather characterized by low air temperature and strong winds [18], [25], [32].

## Materials and Methods

### 3.1 Sampling of water and dissolved gases

Water and dissolved gas sampling was carried out in March 2010 along vertical profiles from the lake surface to the bottom at regular intervals of 5 m (Lake Hule) and 10 m (Lake Río Cuarto), in sites corresponding to the deepest points. Permission to sample in both lakes was guaranteed by Red Sismológica Nacional and Universidad de Costa Rica. According to the *single hose* method [33]–[35], water and dissolved gas samples were collected using a sampling line consisting of 10 m long Rilsan tubes (Φ = 6 mm) connected among them by steel connectors. Once the tube end was lowered to the chosen depth, water was pumped up to the surface through the sampling line using a 150 mL glass syringe equipped with a three-way teflon valve and transferred into plastic bottles after the displacement of a water volume double than the inner volume of the tube. One filtered (0.45 µm) and two filtered-acidified (with ultrapure HCl and HNO_3_, respectively) water samples were collected in polyethylene bottles for the analysis of anions, cations and trace species, respectively. A fourth water aliquot was collected in glass bottles with the addition of HgCl_2_ for the analysis of water isotopes and ^13^C/^12^C ratios of total dissolved inorganic carbon (TDIC). Five hundred mL of water were filtered immediately after the sampling recovery through sterile cellulose mixed esters 0.22 µm pore size filters (GSWP, Millipore, USA) for the analysis of prokaryotic populations. The filters were stored at −20°C in RNAlater solution (Quiagen, Italy), to prevent nucleic acid degradation. Dissolved gases were sampled using pre-evacuated 250 mL glass vials equipped with a Teflon stopcock and connected to the sampling line used to collect water samples. Sampling flasks were filled with water up to ¾ of the inner volume [36]–[38].

### 3.2 Field measurements

Water depth (m), temperature (°C), pH, Eh and electrical conductivity (EC; µS cm^−1^) along the lake vertical profiles were measured using a Hydrolab MiniSonde 5 equipped with a data logger for data storage. The nominal precisions were: depth ±0.05 m; T±0.1°C; pH±0.2; Eh±20 mV; EC±1 µS cm^−1^. Alkalinity was measured *in situ* by acidimetric titration using 0.01 N HCl. The analytical error for alkalinity analysis was ≤5%.

### 3.3 Chemical and isotopic analysis of water and dissolved gases

Main anions (Cl^−^, SO_4_^2−^, NO_3_^−^, Br^−^ and F^−^) and cations (Na^+^, K^+^, Ca^2+^, Mg^2+^, NH_4_^+^ and Li^+^) were analyzed by ion-chromatography (IC) using Metrohm 761 and Metrohm 861 chromatographs, respectively. The analytical error for major water constituents was ≤5%. Trace elements at selected depths were analyzed at the INGV of Palermo by Inductively Coupled Plasma Mass spectrometry (ICP-MS, Agilent 7500-ce). For most of the elements the analytical uncertainty was in the order of 5-10% [39].

The ^18^O/^16^O and ^2^H/^1^H isotopic ratios of water (expressed as δ^18^O-H_2_O and δD-H_2_O ‰ vs. V-SMOW, respectively) from selected depths were analyzed using a Finnigan Delta plusXP continuous-flow mass spectrometer (MS) coupled with a GasbenchII gas-chromatographic device (GBII), according to equilibration techniques with CO_2_ for oxygen [40], and with H_2_ for hydrogen [41]. The analytical uncertainties were ±0.08‰ and ±1‰ for δ^18^O and δD values, respectively.

The ^13^C^/12^C ratios of TDIC (expressed as δ^13^C_TDIC_ ‰ vs. V-PDB) at selected depths were determined on CO_2_ produced by reaction of 3 mL of water with 2 mL of anhydrous phosphoric acid in vacuum [42] using a Finningan Delta Plus XL mass spectrometer. The recovered CO_2_ was analyzed after a two-step extraction and purification procedures of the gas mixtures by using liquid N_2_ and a solid-liquid mixture of liquid N_2_ and trichloroethylene [43], [44]. The analytical uncertainty was ±0.05 ‰.

Dissolved gas composition was calculated using i) the composition of the gas phase stored in the headspace of the sampling glass flasks, ii) the gas pressure in the flask headspace, iii) the headspace volume, and iv) the solubility coefficients in water of each gas compound [45]. The inorganic gas compounds hosted in the flask headspace (CO_2_, N_2_, CH_4_, Ar, O_2_, Ne, H_2_ and He) were determined using a gas-chromatograph (Shimadzu 15a) equipped with a Thermal Conductivity Detector (TCD). Methane was analyzed with a Shimadzu 14a gas-chromatograph equipped with a Flame Ionization Detector (FID). The analytical error for dissolved gas analysis was ≤5%.

The analysis of the ^13^C/^12^C ratios of CO_2_ (expressed as δ^13^C-CO_2_ ‰ vs. V-PDB) stored in the flask headspace (δ^13^C-CO_2STRIP_) of selected samples was carried out with a Finningan Delta S mass spectrometer after standard extraction and purification procedures of the gas mixtures [43], [44]. Internal (Carrara and San Vincenzo marbles) and international (NBS18 and NBS19) standards were used for the estimation of external precision. The analytical uncertainty was ±0.05‰. The ^13^C/^12^C ratio of dissolved CO_2_ (δ^13^C-CO_2_) was calculated from the δ^13^C-CO_2STRIP_ values using the ε_1_ factor for gas-water isotope equilibrium proposed by Zhang *et al.* [46], as follows:
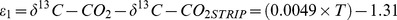
(1)


The analysis of the ^13^C/^12^C and ^2^H/^1^H ratios of dissolved CH_4_ (expressed as δ^13^C-CH_4_ ‰ vs. V-PDB and δD-CH_4_ ‰ vs. V-SMOW, respectively) of selected samples was carried out by mass spectrometry (Varian MAT 250) according to the procedure and the sample preparation described by Schoell [47]. The analytical uncertainty was ±0.15‰.

The ^3^He/^4^He ratios, expressed as R/Ra values, where R is the ^3^He/^4^He isotopic ratio in gas samples and Ra is that of the air equal to 1.39×10^−6^ [48], [49], were determined in selected gas samples stored in the sampling flask headspace at the INGV laboratories of Palermo, using the method described in Inguaggiato and Rizzo [50]. The R/Ra values were corrected for air contamination on the basis of measured He/Ne ratios. The analytical uncertainty was ±0.3%.

### 3.4 Microbiological analysis

DNA extraction for the analysis of microbial populations was performed according to the protocol reported by Mapelli *et al.* [51] and quantified by NanoDrop 1000 spectrophotometer (Thermo Scientific, Waltham, MA). 16S rRNA gene was amplified in PCR reactions using universal primers for bacteria with GC-clamp as described in Marasco *et al.* [52]. Denaturing Gradient Gel Electrophoresis (DGGE), applied to the bacterial 16S rRNA gene amplified from the total water metagenome, was performed by loading DGGE-PCR products (∼150 ng) in a 0.5 mm polyacrylamide gel (7% [w/v] acrylamide-bisacrylamide, 37.5∶1) containing 40 to 55% urea-formamide denaturing gradient, where 100% denaturant corresponds to 7 M urea and 40% [vol/vol] formamide [52]. DGGE profiles were analyzed by using Image J software (available at http://rsb.info.nih.gov/ij/) and cluster analysis was performed using Microsoft Excel XLSTAT software (Addinsoft Inc., New York, NY, USA). DGGE bands were excised from the gel, eluted in water, PCR amplified and sequenced as previously described [52]. The partial 16S rRNA gene sequences obtained from the excised DGGE bands were edited in Chromas lite 2.01 (http://www.technelysium.com.au) and subjected to BLAST search (http://blast.ncbi.nlm.nih.gov/Blast.cgi). The nucleotide sequences were deposited in the EMBL public database under the accession numbers HF930552-HF930593. To test the presence of bacteria involved in anaerobic ammonium oxidation (anammox), the functional gene *hzsA* was amplified using primers hzsA_526F and hzsA_1857R as previously reported [53].

454 pyrosequencing assays were performed by using universal-bacterial primers targeting the variable regions of the 16S rRNA, V1–V3 (27 F mod 5′ – AGRGTTTGATCMTGGCTCAG – 3′; 519 R mod bio 5′ - GTNTTACNGCGGCKGCTG - 3′), amplifying a fragment of approximately 400 bp, and 16S rRNA archaeal primers arch344F (5′ - ACGGGGYGCAGCAGGCGCGA – 3′) and arch915R (5′ - GTGCTCCCCCGCCAATTCCT -3′). The amplified 16S rRNA regions contained enough nucleotide variability to be useful in identification of bacterial and archaeal species [54], [55]. PCR reactions and next generation 454 pyrosequencing were performed at MR DNA laboratories (Shallowater, TX – U.S.A.). A first quality filtering was applied, removing all the sequences that were shorter than 300 bp, longer than 500 bp or with an average quality score under 25. All original and non-chimeric 454 sequences are archived at EBI European Read Archive. The high-quality 16S rRNA gene sequences obtained by 454 pyrosequencing were analysed using QIIME [56]. The sequences were clustered into operational taxonomic units based on a threshold of 97% (OTU_97_) sequence identity, using uclust [57] and one sequence for each OTU_97_, as representative, was aligned to Greengenes (http://greengenes.lbl.gov/) using PyNast [56]. Sequence identification was conducted using Ribosomal Database Project classifier [58], with default parameters. For each sample rarefaction curves of the observed species and of Shannon index were estimated in order to analyse the species sampling coverage. The OTU_97_ diversity within and between sample/s (respectively alpha and beta diversity) was estimated using QIME workflow script alpha_rarefaction.py. Shannon diversity index was calculated by PAST software [59]. Library coverage was calculated for each library using the equation C =  [1– (n1/N)] ×100, where n1 is the number of singleton OTU_97_, and N is the total number of reads in the library. To remove noise from the data, including potential rare contaminants, OTU_97_ not meeting the criterion of being present at least 0.1% of the total number of reads were removed.

## Results

### 4.1 Vertical profiles of temperature, EC, pH and Eh

**Figure 4:**
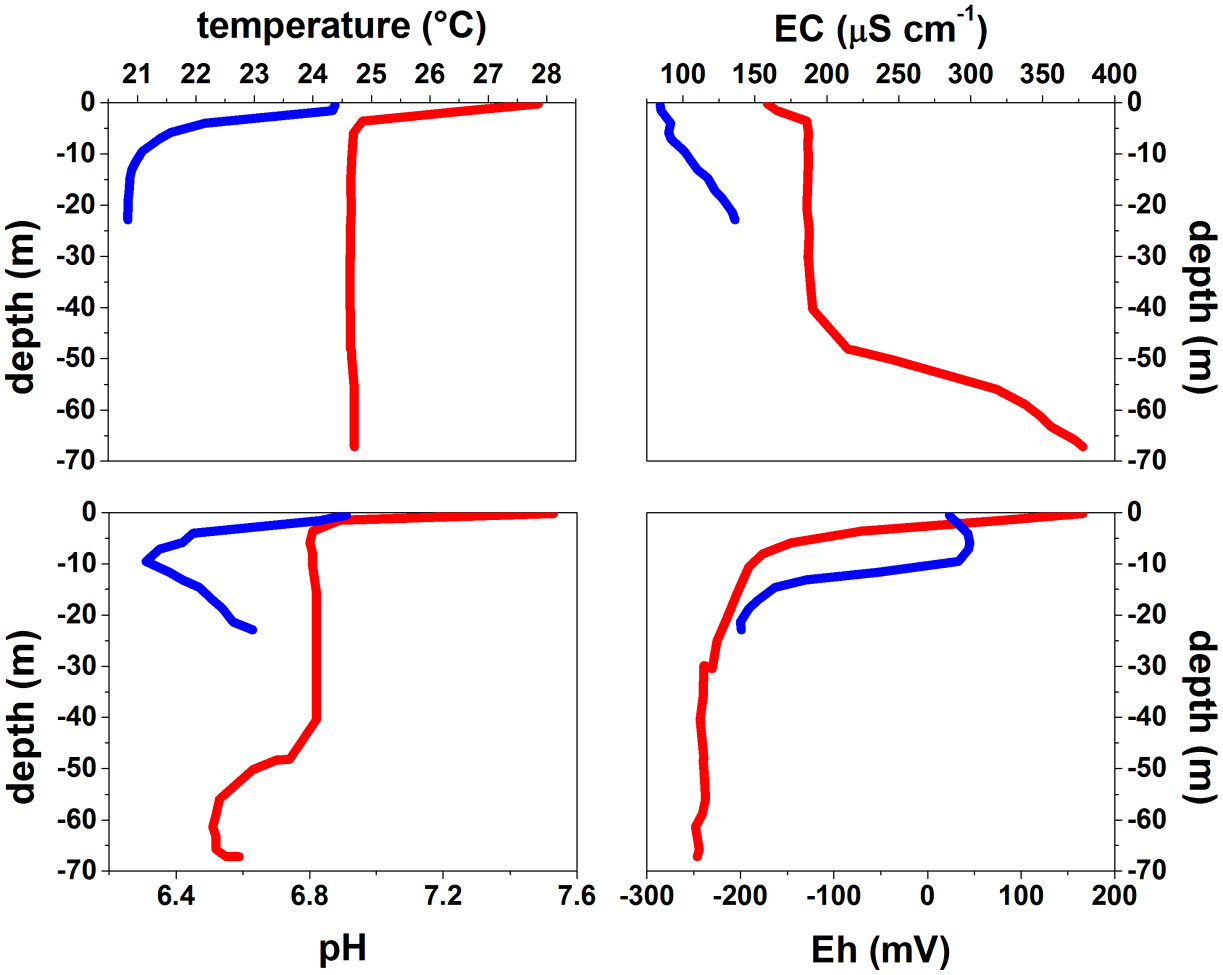
Vertical profiles of temperature (°C, a), electrical conductivity (EC, in µS cm^−1^, b), pH (c), and redox potential (Eh, in mV, d) in Lake Hule (blue line) and Lake Río Cuarto (red line).

Temperature, EC, pH, and Eh along the vertical profiles of the lakes are shown in Tab. 1 and Fig. 4. Both Hule and Río Cuarto lakes showed relatively high temperature at the surface (24.1 and 27.9°C, respectively), and a thermocline at shallow depths (starting from −2.5 and −5 m, respectively), with minimum temperatures of 20.8 and 24.6°C, respectively, at the lake bottoms (Fig. 4a). The temperature profiles were consistent with those reported in previous studies [17], [18], [23], [24], [25], [32], [60], except those of the epilimnion, likely because present and past measurements were carried out in different periods of the year. Lake Hule did not show a clear chemocline, as shown by the EC values that almost constantly increased (from 84 to 140 µS cm^−1^) with depth (Fig. 4b). Conversely, Lake Río Cuarto showed two chemoclines: the first one (from 159 to 186 µS cm^−1^) near the surface and the second one (from 190 to 378 µS cm^−1^) between −40 and −67 m depth. The vertical profile of pH values at Lake Hule exhibited a sharp decrease from 6.9 to 6.3 between the depths of 0 m and 10 m, and an opposite trend below this depth, where pH rose from 6.3 to 6.6 (Fig. 4c). At Lake Río Cuarto the pH values decreased in the shallower water strata (from 7.5 to 6.8) and from −40 to −60 m depth (from 6.8 to 6.5), and slightly increased (up to 6.6) at the lake bottom (Fig. 4c). Eh values at Lake Hule (Fig. 4d) showed a sharp decrease between −10 and −15 m (from 33 to −163 mV) and reached the minimum values at lake bottom (−200 mV), whereas at Lake Río Cuarto it strongly decreased (from +166 at surface to −191 mV) at the depth of 10 m displaying the lowest value (−246 mV) at the lake bottom.

**Table 1:** Depth (m), temperatures (°C), pH, Eh (mV), EC (µS cm^−1^), chemical composition, TDS (total dissolved solids), δD-H_2_O and δ^18^O-H_2_O (expressed as ‰ V-SMOW) and δ^13^C_TDIC_ and δ^13^C_TDICcalc_ (expressed as ‰ V-PDB) values of water samples collected.

Lake	depth	date	T	pH	Eh	HCO_3_^−^	F^−^	Cl^−^	NO_3_^−^	SO_4_^2−^	Ca^2+^	Mg^2+^	Na^+^	K^+^	NH_4_^+^	Fe_tot_	Mn	TDS	δD-H_2_O	δ^18^O-H_2_O	δ^13^C_TDIC_	δ^13^C_TDICcalc_
Hule	0	March-10	24.1	7.0	11	42	0.04	1.2	0.05	2.0	7.0	2.3	2.8	1.5	0.01	0.09	0.003	60	−20.3	−3.8	n.a.	n.a.
	5	March-10	21.8	6.5	33	42	0.04	1.9	0.04	2.1	7.4	2.6	3.1	1.7	0.01	n.a.	n.a.	61	n.a.	n.a.	n.a.	n.a.
	10	March-10	21.1	6.3	23	46	0.04	1.2	0.09	2.2	7.2	2.5	3.5	1.5	0.2	0.03	0.92	66	−19.8	−3.7	−11.8	n.a.
	15	March-10	20.9	6.5	−170	60	0.03	1.8	0.07	1.4	7.5	2.8	3.4	1.5	0.4	n.a.	n.a.	79	−n.a.	n.a.	n.a.	n.a.
	23	March-10	20.8	6.6	−227	61	0.04	1.2	0.07	1.9	8.2	2.7	3.6	1.5	0.3	8.0	0.78	90	−22.5	−3.9	−14.3	−12.2
Rìo Cuarto	0	March-10	27.9	7.5	166	85	0.05	1.8	0.4	1.1	12	5.1	5.7	2.7	1.9	0.02	0.004	116	−20.3	−3.3	−8.3	n.a.
	10	March-10	24.7	6.8	−191	92	0.04	1.9	0.6	0.88	13	5.1	5.5	2.7	2.0	3.4	0.27	127	−19.7	−3.4	−7.9	n.a.
	20	March-10	24.7	6.8	−215	93	0.04	1.7	0.1	0.91	13	5.1	5.5	2.7	2.1	n.a.	n.a.	124	n.a.	n.a.	−7.8	−7.8
	30	March-10	24.7	6.8	−230	93	0.04	1.9	0.03	1.1	13	4.9	5.5	2.7	2.1	n.a.	n.a.	124	n.a.	n.a.	−8.6	−8.8
	40	March-10	24.6	6.8	−243	103	0.05	2.1	0.03	0.95	14	5.0	5.5	2.8	2.4	3.6	0.27	140	−22.2	−3.4	−8.4	−8.9
	50	March-10	24.7	6.6	−239	105	0.05	1.8	0.03	0.67	13	5.1	5.6	2.8	3.3	5.4	0.36	143	−22.6	−3.5	−5.1	−7.3
	60	March-10	24.7	6.5	−245	163	0.06	1.8	0.08	0.51	14	5.6	5.9	3.3	9.0	15	0.63	219	−24.5	−3.7	−3.7	−2.0
	67	March-10	24.7	6.6	−246	179	0.05	1.9	0.09	0.42	15	6.0	6.1	3.5	11	22	0.66	246	−23.6	−3.6	−5.2	−1.6

Ion contents and TDS are in mg L^−1^. n.a.: not analyzed; n.d.: not detected.

### 4.2 Chemical and isotopic composition of water samples

**Figure 5:**
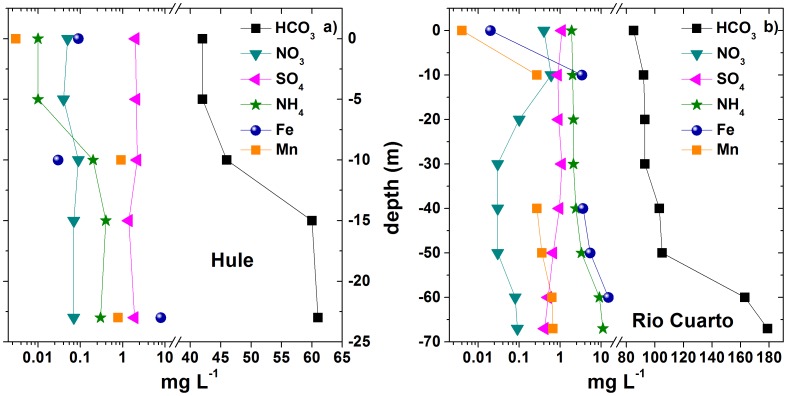
Vertical profiles (in mg L^−1^) of HCO_3_^−^, NO_3_^−^, SO_4_^2−^, NH_4_^+^, Fe_tot_ and Mn in Lake Hule (a) and Lake Río Cuarto (b).

Both lakes showed low TDS values (up to 90 and 246 mg L^−1^, respectively, at lakes bottom) and a Ca^2+^-HCO_3_^−^ composition (Tab. 1). Concentrations of HCO_3_^−^, NH_4_^+^, Fe_tot_ and Mn (Fig. 5a–b) tended to increase towards the two lakes bottom (up to 61 and 179 mg L^−1^, 0.3 and 11 mg L^−1^, 8 and 22 mg L^−1^, 0.9 and 0.7 mg L^−1^ in Hule and Río Cuarto, respectively), whilst oxidized nutrients NO_3_^−^ and SO_4_^2−^, typical electron acceptors in anaerobic environments, showed an opposite behaviour in Lake Río Cuarto, decreasing to 0.03 and 0.4 mg L^−1^, respectively (Fig. 5b). On the contrary, F^−^, Cl^−^, Ca^2+^, Mg^2+^, Na^+^, K^+^ and, only in Lake Hule, NO_3_^−^ and SO_4_^2−^, did not display specific vertical trends along the lakes water column.

The δD-H_2_O values in Hule and Río Cuarto lakes ranged from −26.5 to −20.5 ‰ and −24.4 to −19.7 ‰ V-SMOW, respectively, while those of δ^18^O-H_2_O varied from −4.7 to −4.6 ‰ and from −4.5 to −4.0 ‰ V-SMOW, respectively (Tab. 1). The δ^13^C_TDIC_ values were between −14.3 and −11.8 ‰ and −8.6 to −3.7 ‰ V-PDB, in Hule and Río Cuarto, respectively.

Trace element composition did not differ significantly between the two lakes. The most abundant trace elements (>4 µg L^−1^) along Hule and Río Cuarto vertical profiles were Al, B, Ba, Rb, Sr and Zn. The maximum concentrations of Co, Cu, Ni, Ti and V (<2.2 µg L^−1^) were observed at the bottom layer of Lake Río Cuarto (−67 m) and the other measured trace elements (As, Cd, Cr, Cs, Li, Mo, Pb, Sb, Se, Th, U) were all <1 µg L^−1^ (Tab. 2). In terms of vertical distribution, those trace elements that clearly increased towards both lakes bottom were Al, As, Ba, Co, Ni, Sr, Ti and V (Tab. 2), whilst Mo concentrations showed a decrease with depth only in Lake Río Cuarto.

**Table 2:** Trace elements composition of water samples collected.

Lake	depth	Al	As	B	Ba	Cd	Co	Cr	Cs	Cu	Li	Mo	Ni	Pb	Rb	Sb	Se	Sr	Th	Ti	U	V	Zn
Hule	0	5.4	0.11	5.0	5.1	0.04	<0.05	<0.05	0.04	0.25	0.11	0.11	0.66	0.04	4.6	0.01	0.03	69	<0.02	0.31	<0.02	0.88	3.3
	5	n.a.	n.a.	n.a.	n.a.	n.a.	n.a.	n.a.	n.a.	n.a.	n.a.	n.a.	n.a.	n.a.	n.a.	n.a.	n.a.	n.a.	n.a.	n.a.	n.a.	n.a.	n.a.
	10	5.0	0.12	4.5	9.0	0.08	0.61	<0.05	0.05	0.12	0.11	0.12	0.79	0.04	4.9	0.01	0.02	81	<0.02	0.30	<0.02	0.40	4.4
	15	n.a.	n.a.	n.a.	n.a.	n.a.	n.a.	n.a.	n.a.	n.a.	n.a.	n.a.	n.a.	n.a.	n.a.	n.a.	n.a.	n.a.	n.a.	n.a.	n.a.	n.a.	n.a.
	23	12	0.54	4.5	15	<0.01	1.4	<0.05	0.06	<0.05	0.11	0.16	1.0	0.03	5.4	0.01	0.03	100	<0.02	0.47	<0.02	1.8	1.5
Rìo Cuarto	0	11	0.25	8.8	6.8	0.08	0.16	0.05	0.08	0.38	0.21	0.77	1.0	0.12	8.1	0.17	0.06	118	<0.02	0.66	0.02	0.76	5. 1
	10	7.4	0.21	7.9	20	0.04	0.55	<0.05	0.08	0.10	0.15	0.22	2.8	0.06	8.0	0.03	0.02	115	<0.02	0.44	<0.02	1.0	3. 1
	20	n.a.	n.a.	n.a.	n.a.	n.a.	n.a.	n.a.	n.a.	n.a.	n.a.	n.a.	n.a.	n.a.	n.a.	n.a.	n.a.	n.a.	n.a.	n.a.	n.a.	n.a.	n.a.
	30	n.a.	n.a.	n.a.	n.a.	n.a.	n.a.	n.a.	n.a.	n.a.	n.a.	n.a.	n.a.	n.a.	n.a.	n.a.	n.a.	n.a.	n.a.	n.a.	n.a.	n.a.	n.a.
	40	11	0.21	7.9	22	0.01	0.56	0.06	0.08	0.10	0.14	0.14	1.4	0.15	8.0	0.02	0.02	115	<0.02	0.52	<0.02	1.1	2.8
	50	6.1	0.23	9.1	34	0.01	0.75	<0.05	0.09	0.05	0.15	0.12	1.7	0.04	9.0	0.02	0.02	130	<0.02	0.50	<0.02	1.2	4.5
	60	8.1	0.38	9.6	91	<0.01	1.6	<0.05	0.12	0.10	0.11	0.05	3.1	0.03	10	0.01	0.03	149	<0.02	0.83	<0.02	1.6	3.3
	67	30	0.45	9.3	108	0.02	1.8	0.06	0.12	1.0	0.08	<0.05	3.8	0.22	9.9	0.02	0.04	145	<0.02	1.30	<0.02	2.2	8.4

Chemical concentrations are in µg L^−1^. n.a.: not analyzed.

### 4.3 Chemical and isotopic composition of dissolved gases

**Figure 6:**
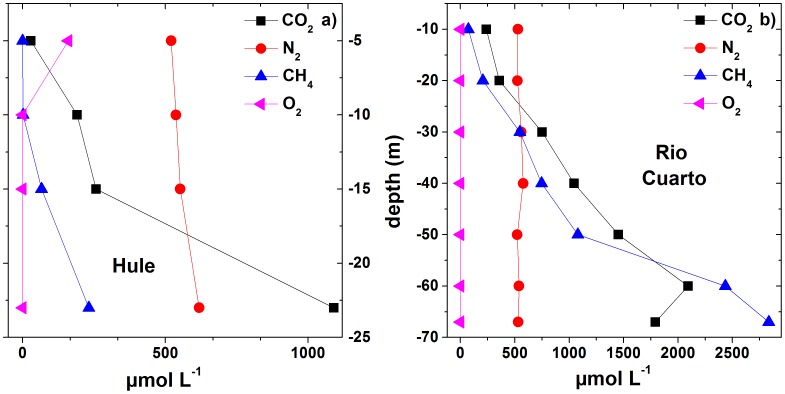
Vertical profiles (in µmol L^−1^) of CO_2_, N_2_, CH_4_ and O_2_ in Lake Hule (a) and Lake Río Cuarto (b).

Molecular nitrogen was the most abundant dissolved gas in the shallow portion of the two lakes (down to the depths of −15 m and −20 m at Lake Hule and Lake Río Cuarto, respectively; Tab. 3). At lower depths CO_2_ dominated the gas composition (up to 1090 and 2090 µmol L^−1^ at Lake Hule and Lake Río Cuarto, respectively), except at the bottom of Lake Río Cuarto (Fig. 6a–b) where CH_4_ concentrations up to 2830 µmol L^−1^ were measured. O_2_ is not present below −10 m depth at Hule and Río Cuarto, defining a clear anaerobic zone (Fig. 6a–b). Ar and Ne did not vary significantly with depth, whereas H_2_ and He concentrations increased with depth in both lakes (up to 0.01 and 0.03 µmol L^−1^ and to 0.04 and 0.3 µmol L^−1^ in Hule and Río Cuarto, respectively; Tab. 3). It is noteworthy to point out that He was an order of magnitude more abundant at Río Cuarto than at Hule. The maximum total pressure (pTOT; Tab. 3) value of dissolved gases was measured at the bottom of Lake Río Cuarto (2.9 atm), whereas pTOT in Lake Hule ranged from 0.79 to 1.1 atm.

**Table 3:** Chemical composition (µmol L^−1^) and total pressure (pTOT; in atm) of dissolved gases (CO_2_, N_2_, CH_4_, Ar, O_2_, Ne, H_2_ and He) and δ^13^C-CO_2_ (expressed as ‰ V-PDB), δ^13^C-CH_4_ (expressed as ‰ V-PDB), δD-CH_4_ (expressed as ‰ V-SMOW) and R/Ra values of gas samples collected.

lake	depth	CO_2_	N_2_	CH_4_	Ar	O_2_	Ne	H_2_	He	pTOT	δ^13^C-CO_2_	δ^13^C-CH_4_	δD-CH_4_	R/Ra	He/Ne
Hule	0	n.a.	n.a.	n.a.	n.a.	n.a.	n.a.	n.a.	n.a.	n.a.	n.a.	n.a.	n.a.	n.a.	n.a.
	5	29	520	n.d.	13	160	0.006	0.007	n.d.	0.88	n.a.	n.a.	n.a.	n.a.	n.d.
	10	191	537	1.7	12	n.d.	0.005	0.005	0.005	0.79	n.a.	n.a.	n.a.	n.a.	1.0
	15	257	552	66	13	n.d.	0.006	0.01	0.008	0.85	n.a.	n.a.	n.a.	n.a.	1.4
	23	1090	618	232	15	n.d.	0.008	0.01	0.03	1.1	−16.2	−62.5	−159	0.95	4.1
Rìo Cuarto	0	n.a.	n.a.	n.a.	n.a.	n.a.	n.a.	n.a.	n.a.	n.a.	n.a.	n.a.	n.a.	n.a.	n.a.
	10	239	528	73	12	0.33	0.006	0.01	n.d.	0.87	n.a.	n.a.	n.a.	n.a.	n.d.
	20	357	524	206	13	n.d.	0.007	0.02	n.d.	0.96	−14.3	−60.7	−233	n.a.	n.d.
	30	751	559	546	13	n.d.	0.007	0.02	0.31	1.3	−14.2	−61.9	−239	n.a.	45
	40	1045	576	746	14	n.d.	0.007	0.02	0.05	1.4	−13.9	−63.8	−241	n.a.	7.6
	50	1450	522	1080	13	n.d.	0.007	0.03	0.09	1.6	−11.6	−72.3	−250	1.15	13
	60	2090	538	2435	13	n.d.	0.006	0.05	0.25	2.6	−6.5	−74.8	−248	n.a.	39
	67	1790	532	2830	13	n.d.	0.007	0.04	0.34	2.9	−6.6	−77.2	−251	1.09	49

Dissolved gas concentrations are in µmol L^−1^. n.a.: not analyzed; n.d.: not detected.

**Figure 7:**
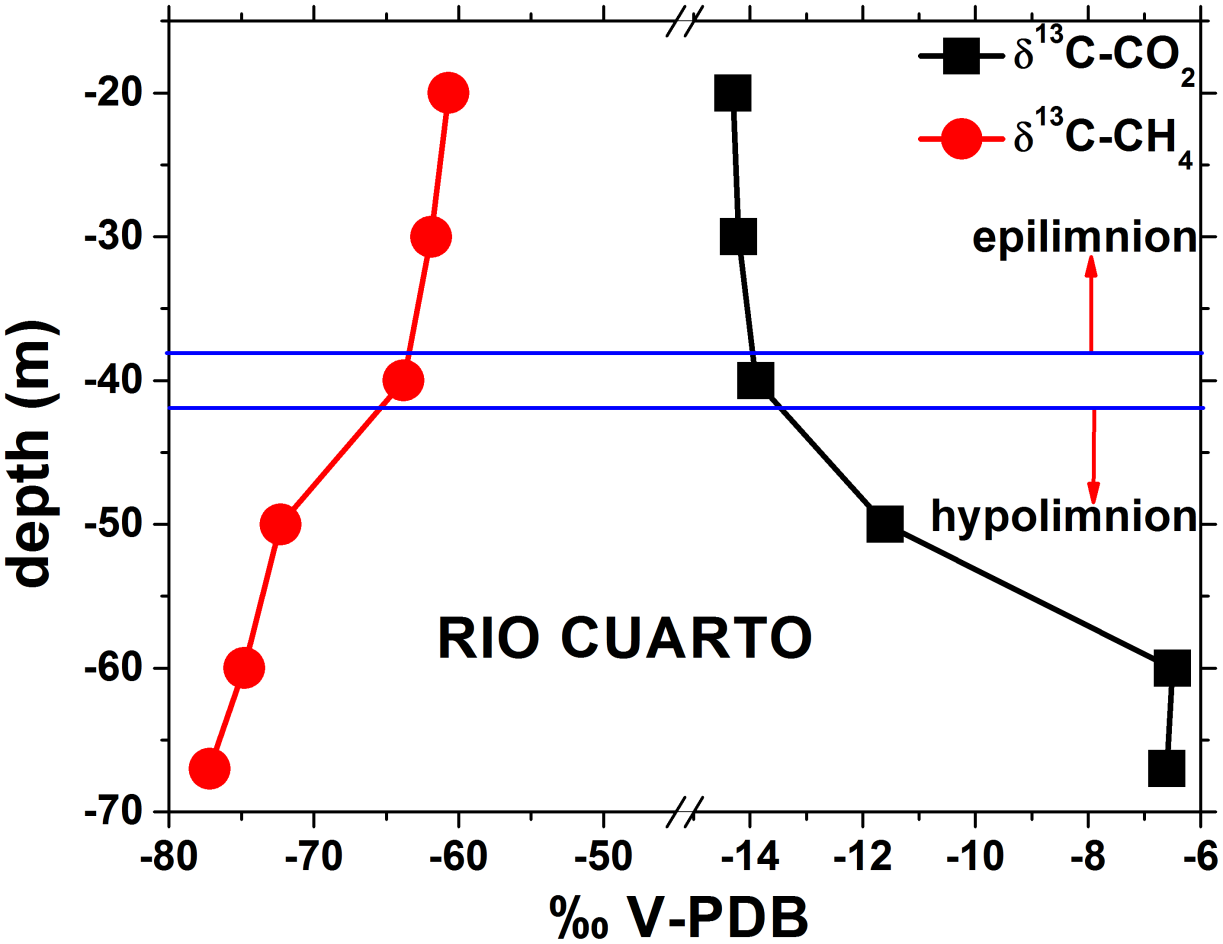
Vertical distribution of δ^13^C-CO_2_ and δ^13^C-CH_4_ of Lake Río Cuarto. See the text for further details.

The δ^13^C-CO_2_ value at the bottom of Lake Hule was −16.2 ‰ V-PDB (Tab. 3). At Lake Río Cuarto, the δ^13^C-CO_2_ values showed an increase with depth, ranging from −14.3 at −20 m to −6.5 ‰ V-PDB at the lake bottom. No specific trends were recognized in the epilimnion (Fig. 7). The δ^13^C-CH_4_ values, basically characterized by the same interval (from −77.2 to −60.7 ‰ V-PDB) in both lakes, showed a rapid decrease in the Río Cuarto hypolimnion. The δD-CH_4_ values of Lake Río Cuarto were significantly more negative (from −251 to −233 ‰ V-SMOW) when compared to that of Lake Hule bottom (−159 ‰ V-SMOW; Tab. 3). The R/Ra values, corrected for the presence of atmospheric helium [61], were 0.95 in Lake Hule (lake bottom) and 1.15 and 1.09 in Lake Río Cuarto (at −50 and −67 m depth, respectively; Tab. 3).

### 4.4 Prokaryotic diversity along the water column

**Figure 8:**
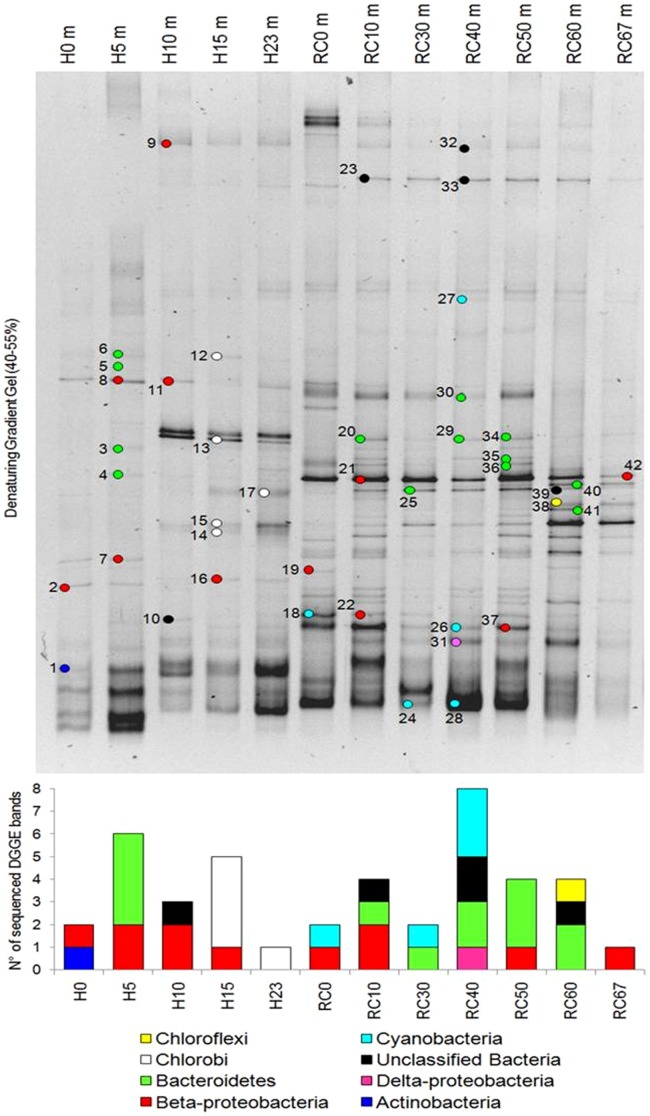
DGGE analysis performed on the bacterial 16S rRNA gene, showing the structure of the bacterial community inhabiting freshwater samples collected from the Hule and Río Cuarto lakes (a); taxonomic identification of bacterial 16S rRNA sequences excised from DGGE bands cut from the Lake Hule and Río Cuarto water profiles (b).

Phylogenetic analyses of 16S rRNA DGGE derived sequences (Fig. 8a–b) allowed to detect 7 phyla within the bacterial communities and to identify the prevalent taxonomic groups colonizing the Hule and Río Cuarto lakes at different depths (Tab. 4). Overall, the sequences were related to uncultured unclassified bacteria previously described in aquatic environments, mainly represented by freshwater of lacustrine origin.

**Table 4:** Phylogenetic identification of the bacterial sequences retrieved from 16S rRNA DGGE gel.

Sample	Phylum	Band	Closest relative	Acc.n°	%	Environments	Closest described specie	Acc. n°	%
Hule 0 m	Actinobacteria	1	Unc. bact.	GU127259	99	Anoxic plant reservoir	*Planktophila limnetica*	FJ428831	95
0 m	Betaproteobacteria	2	Massilia sp.	FJ477729	99	Soil	*Massilia aerilata*	EF688526	96
5 m	Betaproteobacteria	7	Unc. Betaproteobacterium	HM153624	99	Freshwater sample	*Limnobacter thiooxidans*	AJ289885	94
5 m	Betaproteobacteria	8	Unc. Methylophilaceae bact.	HM856563	100	Yellowstone Lake water	*Methylotenera mobilis*	CP001672	95
5 m	Bacteroidetes	5	Unc. Flexibacter sp.	FN668188	98	Lake Zurich water	*Lishizhenia tianjinensis*	EU183317	93
5 m	Bacteroidetes	3	Unc. bact.	EU803667	99	Lake Gatun water	*Mucilaginibacter daejeonensis*	AB267717	83
5 m	Bacteroidetes	4	Unc. bact.	JF295800	97	Soil	*Pedobacter terricola*	EF446147	83
5 m	Bacteroidetes	6	Unc. bact.	HM129930	98	Freshwater	*Lishizhenia tianjinensis*	EU183317	92
10 m	Bacteria	9	Unc. bact.	DQ642387	99	Anoxic freshwater	*Chlorobium phaeovibrioides*	Y08105	82
10 m	Betaproteobacteria	11	Unc. bact.	HQ653799	99	Freshwater	*Methylotenera mobilis*	CP001672	95
10 m	Betaproteobacteria	10	Unc. Undibacterium sp.	GU074344	99	Water sample	*Undibacterium pigrum*	AM397630	96
15 m	Betaproteobacteria	16	Massilia sp.	FJ477729	99	Soil	*Massilia aerilata*	EF688526	97
15 m	Chlorobi	12	Unc. Chlorobi bact.	FJ902335	99	Limestone sinkholes	*Chlorobium clathratiforme*	CP001110	96
15 m	Chlorobi	13	Unc. Chlorobi bact.	FJ902335	99	Limestone sinkholes	*Chlorobium clathratiforme*	CP001110	96
15 m	Chlorobi	14	Unc. bact.	HM228636	99	Riverine alluvial aquifers	*Ignavibacterium album*	AB478415	88
15 m	Chlorobi	15	Unc. bact.	HM228636	98	Riverine alluvial aquifers	*Ignavibacterium album*	AB478415	88
23 m	Chlorobi	17	Unc. bact.	HM228636	92	Riverine alluvial aquifers	*Ignavibacterium album*	AB478415	86
Rìo Cuarto 0 m	Betaproteobacteria	19	Unc. Proteobacterium	GU074082	99	Freshwater	*Burkholderia andropogonis*	AB021422	95
0 m	Cyanobacteria	18	Unc. bact.	GQ091396	99	Freshwater	*Synechococcus rubescens*	AF317076	98
10 m	Betaproteobacteria	21	Unc. bact.	DQ060410	98	Soil enrichment culture	*Methylovorus glucosotrophus*	FR733702	95
10 m	Betaproteobacteria	22	Unc. bact.	GU291353	98	Tropical lakes	*Sulfuritalea hydrogenivorans*	AB552842	94
10 m	Bacteroidetes	20	Unc. bact.	AM409988	98	Profundal lake sediments	*Owenweeksia hongkongensis*	AB125062	88
10 m	Bacteria	23	Unc. Chloroflexi bact.	AB116427	93	Coastal marine sediment	*Ignavibacterium album*	AB478415	82
30 m	Cyanobacteria	24	Unc. Cyanobacterium	FJ844093	99	High mountain lake	*Merismopedia tenuissima*	AJ639891	97
30 m	Bacteroidetes	25	Unc. bact.	FJ437920	97	Green Lake water	*Owenweeksia hongkongensis*	AB125062	88
40 m	Bacteroidetes	29	Unc. bact.	AM409988	98	Profundal lake sediment	*Solitalea koreensis*	EU787448	88
40 m	Cyanobacteria	26	Unc. bact.	HQ653660	97	Shallow freshwater lake	*Cyanobium gracile*	AF001477	96
40 m	Cyanobacteria	27	Unc. bact.	GU305729	99	Oligotrophic lakes	*Cyanobium gracile*	AF001477	98
40 m	Cyanobacteria	28	Unc. bact.	FJ262922	99	Freshwater	*Merismopedia tenuissima*	AJ639891	97
40 m	Deltaproteobacteria	31	Unc. bact.	EF515611	97	Anaerobic bioreactor sludge	*Syntrophobacter pfennigii*	X82875	94
40 m	Bacteria	33	Unc. Chloroflexi bact.	AB116427	93	Coastal marine sediment	*Ignavibacterium album*	AB478415	83
40 m	Bacteria	32	Unc. Chlorobi bact.	GQ390242	98	Low-sulphate lake	*Ignavibacterium album*	AB478415	82
40 m	Bacteroidetes	30	Unc. Haliscomenobacter sp.	HM208523	99	sediment resuspension	*Candidatus Aquirestis calciphila*	AJ786341	99
50 m	Betaproteobacteria	37	Unc. bact.	GU291353	98	Tropical lakes	*Sulfuritalea hydrogenivorans*	AB552842	95
50 m	Bacteroidetes	34	Unc. bact.	AM409988	98	Profundal lake sediment	*Owenweeksia hongkongensis*	AB125062	88
50 m	Bacteroidetes	36	Unc. bact.	FJ612364	99	Dongping Lake Ecosystems	*Sphingobacterium alimentarium*	FN908502	88
50 m	Bacteroidetes	35	Unc. bact.	FJ612364	99	Dongping Lake Ecosystems	*Sphingobacterium alimentarium*	FN908502	88
60 m	Chloroflexi	38	Unc. bact.	JF305756	97	Mature fine tailings	*Dehalogenimonas lykanthroporepellens*	CP002084	86
60 m	Bacteria	39	Unc. bact.	GQ860063	99	PCB-Spiked sediments	*Dehalogenimonas lykanthroporepellens*	CP002084	85
60 m	Bacteroidetes	40	Unc. bact.	FM956124	98	Rice field soil	*Owenweeksia hongkongensis*	AB125062	87
60 m	Bacteroidetes	41	Unc. bact.	FJ437920	97	Freshwater	*Owenweeksia hongkongensis*	AB125062	88
67 m	Betaproteobacteria	42	Unc. bact.	DQ060410	98	Soil enrichment culture	*Methylovorus glucosotrophus*	FR733702	95

The table reports the identification of the dominant bands in the PCR-DGGE fingerprinting profiles marked in Fig. 8. %: percent of identity between the DGGE band sequence and closest relative sequence in GenBank. Acc. N°. Accession number of the closest relative sequence in Genebank. Environment: environment of origin of the closest relative sequence.

At Lake Hule a clear shift in taxa distribution was evaluated, corresponding to the transition at ∼10 m depth of the redox potential from positive to negative. The lake epilimnion was mainly colonized by aerobic heterotrophic Bacteroidetes and Betaproteobacteric while deeper anoxic layers (>10 m depth; Fig. 4d) were inhabited by bacteria belonging to the phylum Chlorobi, comprising anaerobic photoautotrophic bacteria (*Chlorobium clathratiforme* and *Ignavibacterium album*). Bacteroidetes and Betaproteobacteria phyla were also the main components of the bacterial community in Lake Río Cuarto. In this lake the shallower portion (down to the depth of 40 m) was colonized by Cyanobacteria affiliated to the genera *Synechococcus*, *Merismopedia* and *Cyanobium*. Differently from Lake Hule, the more uniform composition of the bacterial community in Lake Río Cuarto can be related to the homogeneity of the redox conditions along the water column, which is negative in all the analyzed layers except at the lake surface (Fig. 4d).

The results of DGGE analysis were taken into account to select a sub-set of samples to gain a deeper insight into the microbiome structure by massive pyrosequencing of bacterial and archaeal 16S rRNA libraries. This high-throughput analysis was applied to 3 samples for each lake (0, 10, 15 m depth from Lake Hule, named H0, H10 and H15, and 30, 50, 60 m depth from Lake Río Cuarto, named RC30, RC50 and RC60). Unfortunately, any archaeal library could not be obtained from sample H0. The number of final reads varied among the samples, similarly to the OTU_97_ number, nonetheless a significant coverage of bacterial and archaeal diversity was reached in all the samples (Tab. 5). The number of OTU_97_ present in the archaeal communities was constant along the water column of Lake Rio Cuarto, while in Lake Hule a significant increase was observed with depth (Tab. 5). In all the samples, Proteobacteria were the most abundant bacterial phylum, with the exception of the water samples collected from Lake Rio Cuarto at 50 and 60 m depths (RC50 and RC60) where Cyanobacteria and Chloroflexi were the prevalent phyla, respectively (Tab. 6). Cyanobacteria were also present at high percentage (29.4%) in the oxic surface water sample in lake Hule (Tab. 6). The phylum Chlorobi was widespread in both the lakes in all the samples characterized by negative Eh values, with significant prevalence at 10 and 15 m depth in Lake Hule (18.5 and 17.6%, respectively). Among Proteobacteria, the Epsilon-subgroup was a minor community component in both lakes and Deltaproteobacteria were more abundant in Río Cuarto, especially in the deeper layers (Tab. 6). Alpha- and Gamma-proteobacteria were differently distributed in the two lakes. The latter were particularly abundant in shallower Hule layers (H10 and H15), while the former were present at high percentages throughout the whole Hule water column (Tab. 6). The class Betaproteobacteria, mainly represented by the *Comamonadaceae* and *Methylophilaceae* families, was abundant at all depths in both the lakes (Tab. 6). In Lake Hule between 12.9 and 22.8% of the bacterial community was represented by sequences belonging to the ACK-M1 cluster of the order Actinomycetales, whose presence in lacustrine habitats was previously reported (Tab. 6) [62]. At the oxic-anoxic interfaces, anaerobic ammonium oxidation (anammox) was indicated as an autotrophic denitrification metabolism co-responsible of nitrogen loss from water environments [63]. The research of bacterial taxa known to be responsible of anammox reaction was performed by amplifying with specific primers the functional gene *hzsA*, encoding for hydrazine synthase and recently proposed as an anammox phylomarker [53]. The PCR amplification showed negative results, confirming that anammox populations are absent at Hule and Rio Cuarto lakes.

**Table 5:** Library coverage estimations and sequence diversity of 16S rRNA.

Sample	N. reads/sample	N. OTU_97_	% Coverage*	Shannon index**
H0 Bacteria	9384	586	0.98	4.21
H10 Bacteria	11115	615	0.98	4.21
H15 Bacteria	15872	1260	0.97	4.98
RC30 Bacteria	32932	3017	0.95	4.60
RC50 Bacteria	13291	1609	0.94	4.37
RC60 Bacteria	13530	1882	0.93	5.08
H10 Archaea	1405	68	0.99	2.76
H15 Archaea	5889	289	0.98	2.08
RC30 Archaea	2429	177	0.96	2.66
RC50 Archaea	2005	172	0.95	2.81
RC60 Archaea	2937	178	0.97	2.34

*Library coverage was calculated as C = 1-n/N, where n is the number of OTU_97_ without a replicate, and N is the total number of sequences.

**Shannon diversity index calculated using PAST.

**Table 6:** List of the taxonomic groups, identified according to the results of the 16S rRNA pyrosequencing, composing the bacterial communities in the freshwater samples collected along the depth profiles of the Hule and Río Cuarto lakes.

PHYLUM/CLASS	ORDER	FAMILY	GENUS	H0	H10	H15	RC30	RC50	RC60
Other	Other	Other	Unknown seq	0.41	0.00	0.21	3.81	6.09	1.12
Other	Other	Other	Uncl. Bacteria	0.00	0.14	0.87	0.43	0.15	0.50
Acidobacteria	Uncl. Acidobacteria	Uncl. Acidobacteria	Uncl. Acidobacteria	0.00	0.13	1.15	0.00	0.00	0.00
Acidobacteria	Holophagales	Holophagaceae	Geothrix	0.00	2.79	3.87	0.00	0.00	0.00
Actinobacteria	Acidimicrobiales	Uncl. Acidimicrobiales	Uncl. Acidimicrobiales	0.25	0.08	0.33	3.55	0.78	0.71
Actinobacteria	Actinomycetales	ACK-M1	Uncl. ACK-M1	22.77	12.90	15.06	2.86	3.20	0.91
Actinobacteria	Actinomycetales	Microbacteriaceae	Candidatus Aquiluna	0.00	0.00	0.00	0.23	0.55	0.04
Actinobacteria	Uncl. Actinobacteria	Uncl. Actinobacteria	Uncl. Actinobacteria	0.00	0.00	0.01	0.21	0.01	1.15
Bacteroidetes	Uncl. Bacteroidetes	Uncl. Bacteroidetes	Uncl. Bacteroidetes	0.00	0.04	0.00	3.78	1.89	4.29
Bacteroidetes	Flavobacteriales	Cryomorphaceae	Uncl. Cryomorphaceae	3.32	0.00	0.00	0.00	0.00	0.00
Bacteroidetes	Flavobacteriales	Flavobacteriaceae	Flavobacterium	0.03	0.00	0.00	0.37	0.05	0.82
Bacteroidetes	Sphingobacteriales	Uncl. Sphingobacteriales	Uncl. Sphingobacteriales	0.00	0.00	0.00	2.19	11.55	0.22
Bacteroidetes	Sphingobacteriales	Chitinophagaceae	Uncl. Chitinophagaceae	2.29	0.42	0.24	0.00	0.00	0.00
Chlorobi	Chlorobiales	Chlorobiaceae	Uncl. Chlorobiaceae	0.00	14.40	10.96	0.12	0.08	0.11
Chlorobi	Ignavibacteriales	Other	Uncl. Ignavibacteriales	0.00	1.39	4.53	0.32	0.72	0.11
Chlorobi	Uncl. Chlorobi	Uncl. Chlorobi	Uncl. Chlorobi	0.00	2.77	2.11	6.71	3.57	5.83
Chloroflexi	Uncl. Anaerolineae	Uncl. Anaerolineae	Uncl. Anaerolineae	0.00	0.05	4.85	1.36	0.38	1.55
Chloroflexi	Uncl. Dehalococcoidetes	Uncl. Dehalococcoidetes	Uncl. Dehalococcoidetes	0.00	0.01	1.00	4.24	1.20	39.52
Cyanobacteria	Uncl. Cyanobacteria	Uncl. Cyanobacteria	Uncl. Cyanobacteria	0.01	0.00	0.01	1.01	2.89	0.07
Cyanobacteria	Synechococcales	Synechococcaceae	Prochlorococcus	29.4	2.56	1.45	17.19	40.20	1.47
OP3	Uncl. OP3	Uncl. OP3	Uncl. OP3	0.00	0.00	0.00	0.27	0.09	0.56
OP8	Uncl. OP8	Uncl. OP8	Uncl. OP8	0.00	0.00	0.00	0.05	0.00	1.20
Planctomycetes	Uncl. Phycisphaerae	Uncl. Phycisphaerae	Uncl. Phycisphaerae	0.00	0.00	0.00	0.36	0.15	0.14
Planctomycetes	Gemmatales	Gemmataceae	Uncl. Gemmataceae	0.16	0.06	0.67	0.65	0.76	0.87
Planctomycetes	Pirellulales	Pirellulaceae	Uncl. Pirellulaceae	0.57	0.39	0.72	2.06	1.03	3.39
Alphaproteobacteria	Rhizobiales	Methylocystaceae	Methylosinus	0.07	0.12	0.47	0.49	0.14	0.09
Alphaproteobacteria	Rhodospirillales	Rhodospirillaceae	Uncl. Rhodospirillaceae	0.04	0.97	1.14	0.08	0.04	0.01
Alphaproteobacteria	Rickettsiales	Uncl. Rickettsiales	Uncl. Rickettsiales	18.31	6.95	14.40	0.30	0.29	0.09
Betaproteobacteria	Uncl. Betaproteobacteria	Uncl. Betaproteobacteria	Uncl. Betaproteobacteria	0.00	0.00	0.00	0.58	0.23	0.36
Betaproteobacteria	Burkholderiales	Burkholderiaceae	Uncl. Burkholderiaceae	0.73	0.08	0.04	0.16	0.26	0.00
Betaproteobacteria	Burkholderiales	Comamonadaceae	Uncl. Comamonadaceae	3.22	0.29	0.44	0.02	0.02	0.00
Betaproteobacteria	Burkholderiales	Comamonadaceae	Limnohabitans	5.18	0.51	0.33	0.00	0.00	0.00
Betaproteobacteria	Burkholderiales	Comamonadaceae	Rhodoferax	4.59	0.12	0.24	0.00	0.00	0.00
Betaproteobacteria	Burkholderiales	Comamonadaceae	Rubrivivax	0.03	6.26	1.22	0.02	0.03	0.00
Betaproteobacteria	Burkholderiales	Oxalobacteraceae	Uncl. Oxalobacteraceae	2.52	0.94	0.86	0.01	0.00	0.00
Betaproteobacteria	Burkholderiales	Oxalobacteraceae	Polynucleobacter	0.67	0.25	0.14	0.07	0.15	0.00
Betaproteobacteria	Methylophilales	Uncl. Methylophilales	Uncl. Methylophilales	0.70	1.01	0.32	0.00	0.00	0.00
Betaproteobacteria	Methylophilales	Methylophilaceae	Uncl. Methylophilaceae	0.03	4.21	2.72	35.99	11.75	20.57
Betaproteobacteria	Rhodocyclales	Rhodocyclaceae	Uncl. Rhodocyclaceae	0.00	1.05	0.36	0.91	2.13	0.09
Deltaproteobacteria	Desulfuromonadales	Geobacteraceae	Geobacter	0.00	0.00	0.01	0.24	0.04	0.66
Deltaproteobacteria	Myxococcales	Uncl. Myxococcales	Uncl. Myxococcales	0.00	0.00	0.98	0.07	0.03	0.06
Deltaproteobacteria	Spirobacillales	Uncl. Spirobacillales	Uncl. Spirobacillales	0.10	0.01	0.04	1.65	3.55	0.67
Deltaproteobacteria	Syntrophobacterales	Syntrophobacteraceae	Syntrophobacter	0.00	0.00	0.00	0.96	0.62	6.96
Epsilonproteobacteria	Campylobacterales	Helicobacteraceae	Sulfuricurvum	0.00	0.00	1.86	1.15	1.91	0.07
Gammaproteobacteria	Uncl. Gammaproteobacteria	Uncl. Gammaproteobacteria	Uncl. Gammaproteobacteria	0.00	0.00	0.00	0.44	1.72	0.10
Gammaproteobacteria	Enterobacteriales	Enterobacteriaceae	Uncl. Enterobacteriaceae	0.17	0.64	0.05	0.11	0.00	0.02
Gammaproteobacteria	Legionellales	Legionellaceae	Uncl. Legionellaceae	0.00	0.05	0.02	0.45	0.07	0.05
Gammaproteobacteria	Methylococcales	Crenotrichaceae	Crenothrix	0.00	17.25	3.37	0.00	0.00	0.00
Gammaproteobacteria	Methylococcales	Methylococcaceae	Methylocaldum	0.07	1.24	2.76	2.31	0.67	4.38
Gammaproteobacteria	Methylococcales	Methylococcaceae	Methylomonas	0.01	0.24	1.12	0.00	0.00	0.00
Gammaproteobacteria	Pseudomonadales	Pseudomonadaceae	Pseudomonas	0.01	16.31	16.47	0.00	0.02	0.02
Gammaproteobacteria	Xanthomonadales	Sinobacteraceae	Uncl. Sinobacteraceae	0.83	0.39	0.02	0.02	0.03	0.01
Verrucomicrobia	Opitutales	Opitutaceae	Opitutus	0.89	2.09	2.50	0.00	0.01	0.00
Verrucomicrobia	Uncl. Opitutae	Uncl. Opitutae	Uncl. Opitutae	2.61	0.89	0.06	0.00	0.04	0.00
Verrucomicrobia	Uncl. Verrucomicrobia	Uncl. Verrucomicrobia	Uncl. Verrucomicrobia	0.00	0.00	0.03	1.52	0.72	0.69
WS3	Uncl. WS3	Uncl. WS3	Uncl. WS3	0.00	0.00	0.00	0.64	0.22	0.47

Uncl: unclassified. Results are expressed as % of the sequences.

As far as the archaeal community is concerned, Euryarchaeota were the most abundant phylum in Lake Rio Cuarto (up to 99%). Methanomicrobia were the most abundant class within this phylum, encompassing in particular the orders *Methanomicrobiales* and *Methanosarcinales* (Tab. 7). Lake Hule showed a different archaeal community, being dominated by Parvarchea and Micrarchaea, with significant concentrations of Crenarchaeota (8.1 and 13.7% at 10 and 15 m depth, respectively), and a minor percentage of Methanomicrobia and unknown taxa (Tab. 7).

**Table 7:** List of the taxonomic groups, identified according to the results if the 16S rRNA pyrosequencing, composing the archaeal communities in the freshwater samples collected along the depth profiles of the Hule and Río Cuarto lakes.

PHYLUM	CLASS	ORDER	FAMILY	GENUS	H10	H15	RC30	RC50	RC60
Unknown seq.	Unknown seq.	Unknown seq.	Unknown seq.	Unknown seq.	2.30	0.00	0.00	0.00	0.00
Uncl. Archaea	Uncl. Archaea	Uncl. Archaea	Uncl. Archaea	Uncl. Archaea	0.00	0.00	0.56	0.34	0.00
Crenarchaeota	MCG	Uncl. MCG	Uncl. MCG	Uncl. MCG	1.63	0.76	0.10	0.68	0.80
Crenarchaeota	MCG	pGrfC26	Uncl. pGrfC27	Uncl. pGrfC27	6.51	12.94	0.29	0.24	0.04
Euryarchaeota	Uncl. Euryarchaeota	Uncl. Euryarchaeota	Uncl. Euryarchaeota	Uncl. Euryarchaeota	0.95	0.30	0.00	0.00	0.00
Euryarchaeota	Methanomicrobia	Uncl. Methanomicrobia	Uncl. Methanomicrobia	Uncl. Methanomicrobia	0.00	0.00	0.10	0.43	0.18
Euryarchaeota	Methanomicrobia	Methanomicrobiales	Methanoregulaceae	Uncl. Methanoregulaceae	0.50	0.32	0.62	1.21	0.55
Euryarchaeota	Methanomicrobia	Methanomicrobiales	Methanoregulaceae	Methanoregula	4.04	5.52	20.39	39.39	7.73
Euryarchaeota	Methanomicrobia	Methanosarcinales	Methanosaetaceae	Methanosaeta	1.01	0.92	46.67	39.59	86.48
Euryarchaeota	Cand. Micrarchaea	Cand. Micrarchaeles	Uncl. Micrarchaeles	Uncl. Micrarchaeles	25.01	73.09	29.84	14.32	3.72
Euryarchaeota	Cand. Parvarchaea	Cand. WCHD3-30	Uncl. WCHD3-30	Uncl. WCHD3-30	30.57	5.09	0.49	2.12	0.15
Euryarchaeota	Cand. Parvarchaea	Cand. YLA114	Uncl. YLA114	Uncl. YLA114	27.48	1.05	0.95	1.69	0.36

Uncl: unclassified. Cand: Candidatus. Results are expressed in % with respect to the total archaeal community.

## Discussion

### 5.1 Processes controlling the water chemistry

**Figure 9:**
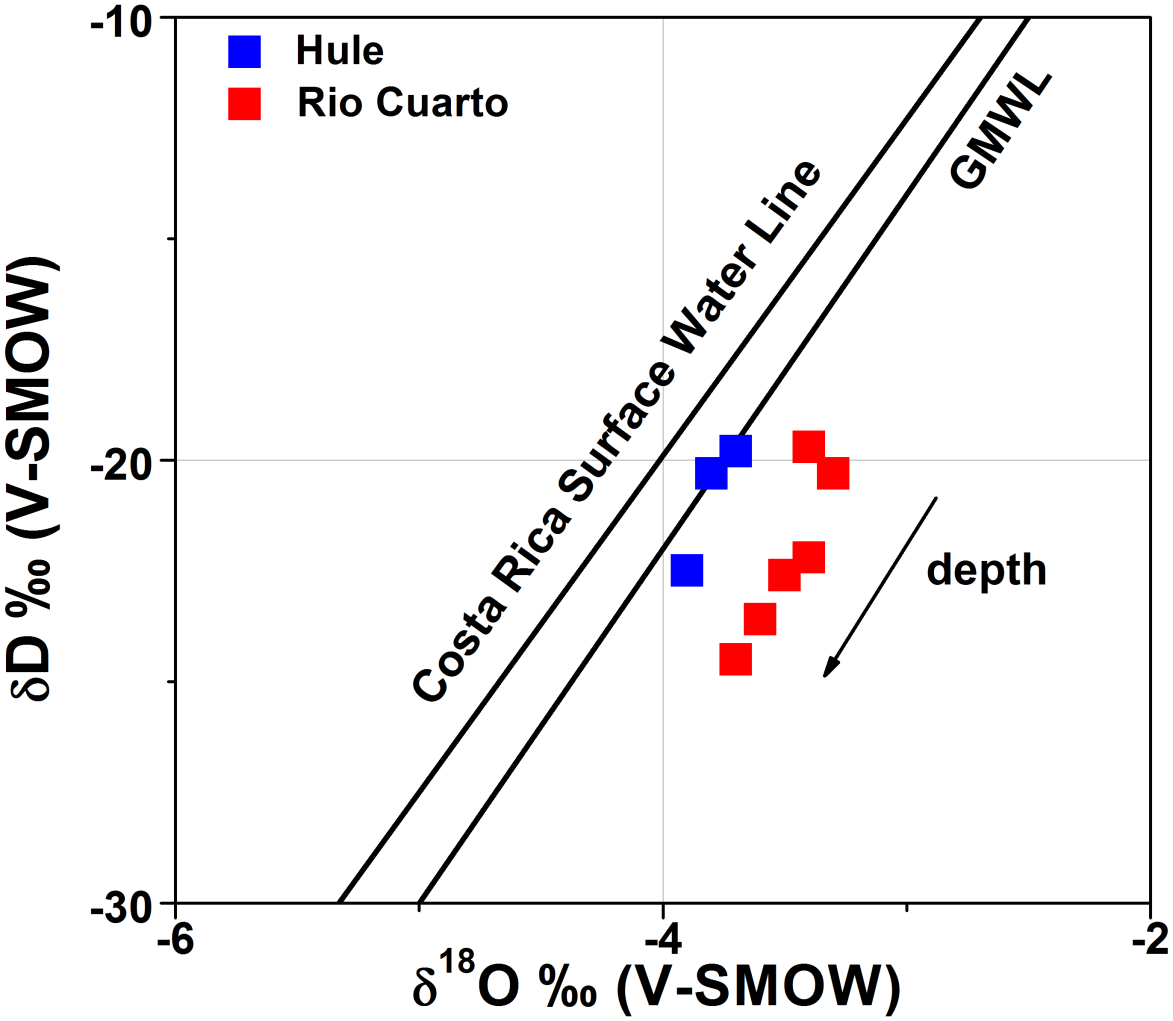
δ^18^O–δD diagram for the water samples from Lake Hule (blue squares) and Lake Río Cuarto (red squares). See the text for details.

Water isotopes can provide notable information on physical-chemical processes affecting the chemistry of volcanic lakes, such as evaporation, water-rock interaction and hydrothermal/meteoric inputs [64]. As shown in Fig. 9, water samples plot near the Global Meteoric Water Line (GMWL) [65] and the Costa Rica Surface Water Line [66], indicated that in both lakes the main water source is meteoric, consistently with their Ca^2+^(Mg^2+^)-HCO_3_^−^ composition, which is typical for superficial waters and shallow aquifers worldwide [67]. Both lakes show a slight D- and ^18^O- depletion at increasing depth, likely related to evaporation affecting epilimnetic waters [64], [68], [69].

The parallel increases of HCO_3_^−^ (Fig. 5) and dissolved CO_2_ (Fig. 6) along the vertical profiles suggest that the behaviour of these two chemical species is controlled by the following reactions:
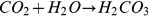
(2)and



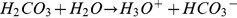
(3)


The observed weak decreases of SO_4_^2−^ and NO_3_^−^ concentrations (Fig. 5) with depth possibly result from microbial activity occurring at anaerobic conditions. The lack of free oxygen in the hypolimnion is favorable for nitrate reduction by microbial denitrification, a typical process in anoxic water bodies [70]–[74]. The genus *Pseudomonas* is known to include denitrifier species [75] and was retrieved at high abundance in pyrosequencing libraries in the anoxic layers of Lake Hule, constituting up to 16% of the total bacterial community (Tab. 6). In the Hule anoxic layers, 16S rRNA pyrosequencing allowed to detect additional denitrifying genera like *Sulfuricurvum, Opitutus* and *Geothrix* (Tab. 6). Sulfate reducing bacteria (SRB) of the genus *Syntrophobacter* were retrieved by 16S rRNA pyrosequencing in the deepest layers of the Río Cuarto water column (Tab. 6), and could be responsible of the weak depletion observed for SO_4_^2−^ (Fig. 5b). Nevertheless, the relatively low SO_4_^2−^ and NO_3_^−^ concentrations, typical of meteoric-sourced lakes, implies that sulfate reduction and denitrification have a minor impact on the chemistry of the two lakes. The increase of NH_4_^+^ concentrations with depth (Fig. 5) is apparently suggesting direct NH_4_^+^ production within the hypolimnion via ammonification processes [76].

The increase of Fe and Mn contents in the deepest water layers can be attributed to direct production inside the bottom sediments by minerogenic processes [77]–[79], although their presence as solutes is limited by the formation of insoluble Fe- and Mn-hydroxides. Göcke [24] suggested that the high concentration of Fe in Lake Hule is also caused by the addiction of yellow/brownish Fe(OH)_3_ material through the southern brooklets, which subsequently precipitates in the hypolimnion and iron is reduced to the ferrous state, as also supported by the relatively low Eh values (Fig. 4). Oxidation of hypolimnetic Fe^2+^ in the epilimnion would explain the yellow-reddish color of the shallow water layer that was occasionally observed in these lakes as a consequence of water rollover [18], [25]. Nevertheless, the red coloration observed at Lake Hule in February 1991 was likely caused by the presence of dense purple clumps or masses floating of *Merismopedia* [18], a genus belonging to the phylum Cyanobacteria that were observed by DGGE at −30 and −40 m depth in Lake Río Cuarto (Tab. 4).

**Figure 10:**
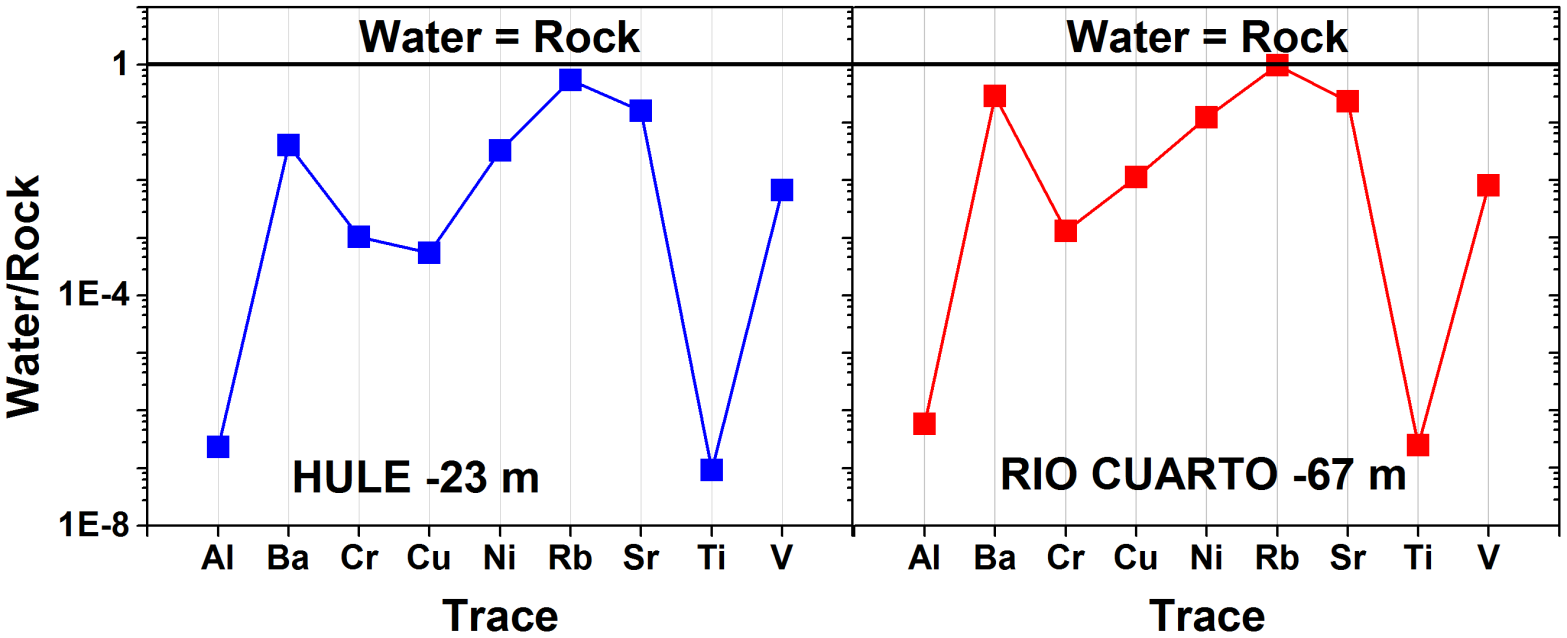
Spider-diagrams, where concentrations of selected trace elements in Lake Hule (a) and Lake Río Cuarto (b) maximum depths are normalized to those measured in basalt rock samples collected from the young intra-caldera cone at Laguna Hule [80].

As shown in the spider-diagrams of Fig. 10, where concentrations of Al, Ba, Cr, Cu, Ni, Rb, Sr, Ti and V at maximum depths for both lakes are normalized to those measured in basalt rock samples collected from the young intra-caldera cone at Laguna Hule (the only one available) [80], water-rock interactions efficiently mobilized soluble elements such as Ba, Rb and Sr, whereas Al and Ti were basically retained in the rock matrix. In particular, Cr and Ni, as well as As and Co, are possibly related to the dissolution of Mn-and Fe-oxide particles that settled through the chemocline [78], [81], [82]. The concentrations of dissolved V are strongly correlated with those of Fe, similarly to what observed for Mo and Mn [29], [83], likely because they belong to the same mineralogical paragenesis. For what concerns the other trace elements, Cu and Zn may be related to dissolution of stable organic complexes buried in the bottom sediments [29]. Cs, Rb and B, which are strongly correlated with Li (Tab. 2), can be considered as conservative elements, likely due to the strong affinity of alkali ions and boric acid for the aqueous phase [82]. The relatively low Mo concentrations at increasing depth in Lake Río Cuarto (Tab. 2) may be related to its consumption during microbial nitrate reduction [29].

### 5.2 Processes governing chemical and isotopic composition of dissolved gases

#### 5.2.1 Noble gases, N_2_, O_2_, and H_2_

Dissolved gas species in volcanic lakes basically originate from i) external sources (e.g. atmosphere, volcanic-hydrothermal fluids) and/or ii) microbial activity occurring both in lake water and at water-sediment interface [4], [14], [28], [84], [85], [86].

Dissolved Ar and Ne in lakes are related to air dissolution through the lake surface, a process that is mainly controlled by atmospheric pressure and the water temperature [87]. The inert noble gases behave inertly in any bio-geochemical process and thus along the lake water column they are affected by advection and diffusion. Accordingly, Ar and Ne concentrations in the two investigated lakes did not show significant variations with depth (Tab. 3). Conversely, O_2_, which is typically consumed by aerobic microbial populations for oxidation of organic matter and reduced ionic species, rapidly decreases with depth, and was virtually absent at depths ≥5 and 10 m, in Hule and Río Cuarto lakes, respectively. It is worth noting that the N_2_/Ar ratios were slightly higher than that of air saturated water (∼40), suggesting the addition of N_2_ from an extra-atmospheric source. This hypothesis is expected to be confirmed by δ^15^N values that are presently not available, although the relatively high N_2_/Ar ratios are apparently consistent with nitrate depletion with depth and microbial denitrification in both lakes. Consistently with the N_2_ excess, the distribution of N_2_ concentrations in both lakes showed significant variations with depth (Tab. 3), probably related to N_2_ production and consumption by denitrifiers and nitrogen fixing prokaryotes, respectively. Microbial N_2_ fixation, depending on light [88] and the presence of bio-available trace metals [89], can be carried out by heterocyst-forming species in water and in sediment pores [90]–[92]. *Cyanobacteria* were indeed retrieved by both DGGE and pyrosequencing in surface layers of Río Cuarto and Hule lakes (where they constitute 26% of the total bacterial community in the oxic layer H0, Tab. 6), supporting the occurrence of N_2_ fixation in both the lakes.

H_2_ increase with depth in the hypolimnion at Hule and Río Cuarto (Tab. 3) suggests a production of H_2_ likely related to fermentation of organic matter under anaerobic conditions at the water-sediment interface. Additionally, photoreactions carried out by *Cyanobacteria*, abundantly present in the Río Cuarto deep layers and in the upper layer of the Hule water columns (Tab. 6), could be responsible of H_2_ production [93]–[97]. Once produced at the lake bottom, H_2_ can be consumed acting as electron donor for hydrogenotrophic methanogenic archaea and SRB [98]–[100], detected in Río Cuarto pyrosequencing libraries. Moreover, it slowly diffuses up to shallower, oxygenated layers where it can be consumed by hydrogen-oxidizing bacteria [101]–[104].

The presence of an extra-atmospheric source for helium can be recognized on the basis of the R/Ra values (Tab. 3), which are relatively high (up to 20 or more) for mantle gases, and as low as 0.01 in fluids from crustal sources [61]. Dissolved gas samples from Hule and Río Cuarto lakes showed R/Ra values ∼1 that, coupled with the relatively high He/Ne ratios (49 and 4.1 at Lake Rio Cuarto and Lake Hule, respectively), indicate a significant fraction of mantle He, whose uprising is likely favored by the fault system characterizing this area [18].

#### 5.2.2 CO_2_ and CH_4_

CO_2_ and CH_4_ are the most abundant extra-atmospheric dissolved gases present in Hule and Río Cuarto lakes. As already mentioned, dissolved CO_2_ controls pH values and HCO_3_^−^ concentrations. Previous studies [17], [18], [20], [22], [23] have hypothesized that these lakes are affected by CO_2_ inputs through the bottom, as supported by the presence of CO_2_-rich bubbling pools and caverns or boreholes with high CO_2_ concentrations characterizing this area [18], [105]. A significant contribution of mantle CO_2_ is indicated by the δ^13^C-CO_2_ value of the dissolved gas sample collected at the maximum depth of Lake Río Cuarto (−6.6 ‰ vs. V-PDB; Tab. 3), which is in the range of mantle gases (from −8 to −4 ‰ vs. V-PDB) [106]. Although not confirmed by the δ^13^C-CO_2_ values, the CO_2_/CH_4_ ratio measured in the dissolved gas at the bottom of Lake Hule (4.7) is too high, even higher than that of Río Cuarto bottom sample (0.63), to be ascribable to microbiological processes. This would imply that even at Lake Hule a strongly negative isotopic signature of CO_2_ is externally added to the bottom waters, possibly from a CO_2_-rich source deriving from oxidation of previously produced hydrocarbons.

The δ^13^C-CO_2_ values at the bottom of Lake Hule (−16.2 ‰ vs. V-PDB) and at depths between −20 and −50 m in Lake Río Cuarto (as low as −14.3 ‰ vs. V-PDB; Tab. 3) were intermediate between those generated by organic matter degradation [24] and mantle degassing [107]–[109], indicating that along the vertical profiles of both lakes, excluding the bottom layers, biogenic processes are the most important sources of CO_2_.

**Figure 11:**
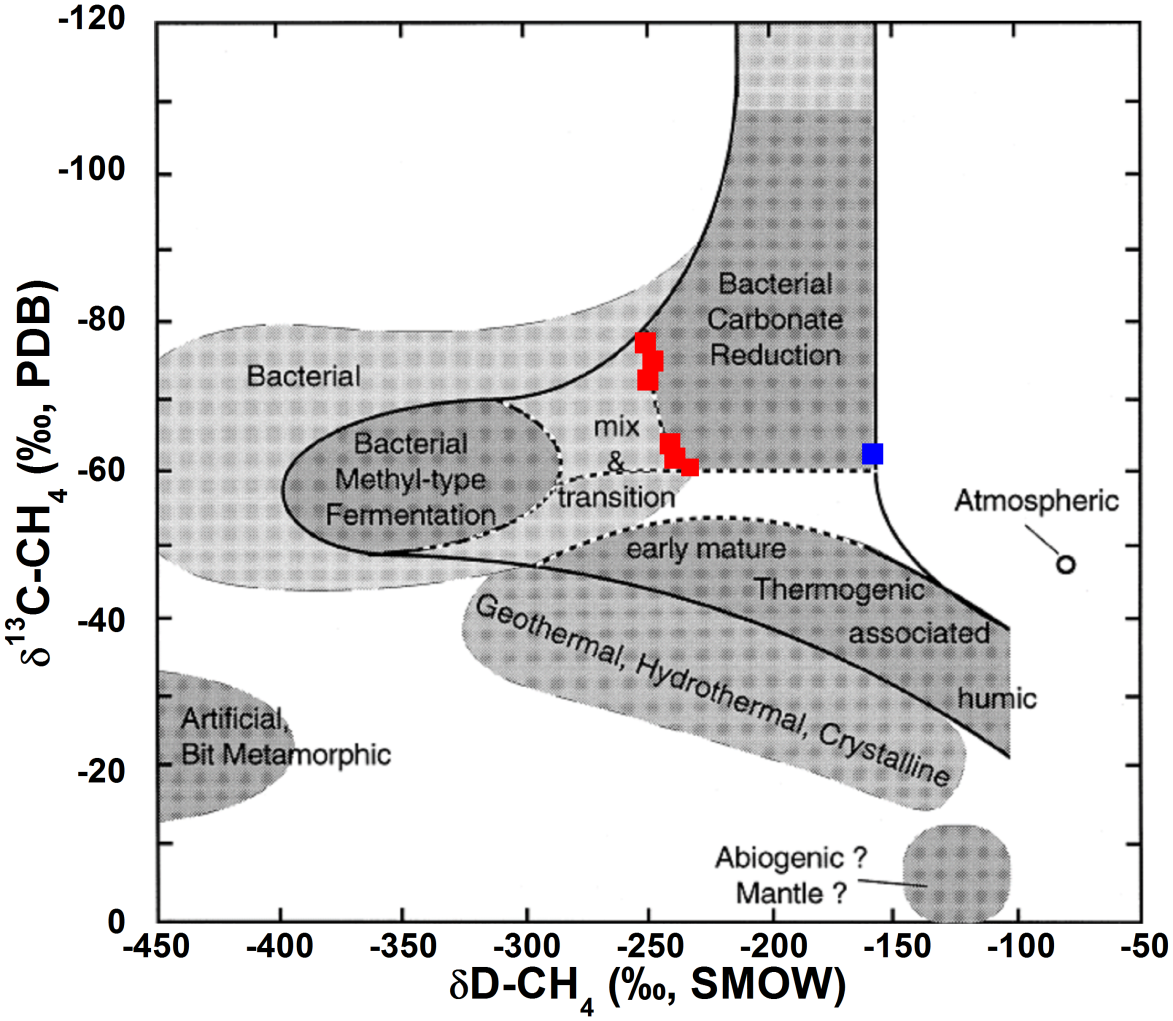
δ^13^C-CH_4_ vs. δD-CH_4_ plot (modified after Whiticar [110]) of Lake Hule (blue square) and Lake Río Cuarto (red squares). See the text for further details.

According to the classification proposed by Whiticar [110], the δ^13^C-CH_4_ and δD-CH_4_ values of the Hule and Río Cuarto lakes indicate that CH_4_ has a biogenic origin (Fig. 11). The vertical profiles of the concentrations and δ^13^C values of CO_2_ and CH_4_ of Lake Río Cuarto (Fig. 7) were thus produced by the combination of different processes occurring at various depths in the lake:

1. At the bottom of the lake, CO_2_ inputs from a deep source likely related to the hydrothermal fluid circulation [18], [111] promote methanogenic processes that have their maximum efficiency within the sediments. Methanogenesis takes place through i) CO_2_ reduction and ii) degradation of organic matter through acetate fermentation [47], [110], [112]–[115]. These processes can be described by the following reactions:
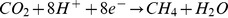
(4)and
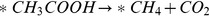
(5)where the * indicates the intact transfer of the methyl position to CH4.
2. In the hypolimnion, microbial CH_4_ production is still active, although this process is accompanied by CO_2_ dissolution, CH_4_ oxidation, and vertical diffusion of both the gas species. Moreover, in correspondence of aerobic/anaerobic boundaries, anaerobic decomposition of organic matter [116]–[118], and CH_4_ oxidation carried out by methanotrophic bacteria can efficiently produce CO_2_ in lakes [86], [119]–[121].
3. In the epilimnion, photosynthetic microorganisms (e.g. *Cyanobacteria*) convert light into biochemical energy through oxygenic photochemical reactions combined with CO_2_ assimilative reduction. Vertical water circulation favors the activity of photosynthetic and methanotrophic bacterial populations, as well as the continuous addition of atmospheric gases from the lake surface.

These hypotheses were confirmed by the 16S rRNA pyrosequencing of samples collected along the water column of Lake Rio Cuarto, demonstrating that archaeal communities encompass almost exclusively methanogenic populations (Tab. 7) typical of freshwater ecosystems, namely *Methanomicrobiales* and *Methanosarcinales* [122]–[124], as also observed in freshwater meromictic lake sediments [125]. *Methanosarcinales* included solely the acetate-utilizing methanogen *Methanosaeta*, the most abundant archaeal genus along the Río Cuarto water column. Within the H_2_-CO_2_ utilizing methanogens of the order *Methanomicrobiales*, *Methanoregula* was the prevalent genus, but unclassified *Methanomicrobiales* and *Methanoregulaceae* sequences were also detected (Tab. 7).

The lack of isotopic data along the vertical profile of Lake Hule did not allow to investigate in detail the (bio)-geochemical processes controlling the vertical profiles of CO_2_ and CH_4_. In this lake the majority of the archaeal 16S rRNA sequences were affiliated within unclassified Euryarchaeota, showing high similarity with the Candidate divisions Micrarchaea and Parvarchaea (Tab. 7) previously described by metagenomics studies of an acidic ecosystem by Baker *et al.* [126], [127]. These archaeal sequences belong to the ARMAN (Archaeal Richmond Mine Acidophilic Nanoorganisms) lineages, which are among the smallest cellular life forms known [126], still poorly described from an ecological perspective. The presence of novel uncultivated lineages in the Lake Hule water is linked to neither specific metabolism nor the influence on the water and dissolved gas chemistry. However, besides a minor fraction of known acetotrophic methanogenic *Methanosarcinales* (Tab. 7), the archaeal community of Lake Hule included also the Miscellaneous Crenarchaeota Group (MCG), within the phylum Crenarchaeota (Tab. 7). MCG is a cosmopolitan clade that was previously detected in both freshwater [128] and marine ecosystems [129], where it had been hypothesized to have a significant role in dissimilatory methane oxidation [129]. This hypothesis leads to the speculation that MCG could have the same ecological function also in the Lake Hule. It is worth noting that the minor percentage of known methanogenic archaea in Lake Hule compared to that of Lake Río Cuarto corresponds to the differences between the lakes in CH_4_ concentrations (Tab. 3).

16S rRNA pyrosequencing of bacterial communities showed that type I and type II methanotrophic bacteria, belonging to the Gamma- (i.e. *Methylocaldum, Methylomonas*, *Crenothrix*) and Alpha-subgroup of proteobacteria (i.e. *Methylocystaceae*) [125], [130], respectively, were abundant in the anoxic layers of Hule and Río Cuarto (Tab. 6), suggesting a key role in the carbon cycle. Within the Beta-proteobacteria, additional families that encompass methylotrophic bacteria, namely *Methylophilaceae*, *Rhodocyclaceae*, and *Comamonadaceae* [131], [132], were retrieved by deep sequencing in the same water layers both in Lake Hule and Lake Río Cuarto, the latter hosting up to 36% of *Methylophilaceae* at 30 m depth (Tab. 6). Within the family *Comamonadaceae*, relevant in Lake Hule, 5.2% of the bacterial sequences from the surface layer were affiliated to the genus *Limnohabitans*, which was reported to play a functional key role in freshwater habitats and showing high ecological diversification [133]. Moreover, 6.3% of the bacterial sequences were affiliated to the genus *Rubrivivax* that includes, among the few characterized species, strains able to oxidize carbon monoxide producing carbon dioxide and hydrogen [134]. The presence of the genus *Syntrophobacter* at 60 m depth in Río Cuarto (RC60) is in agreement with the establishment in deep anoxic layers of syntrophic relations between organic acid degrading bacteria and methanogenic archaea. Members of this genus were commonly detected in anaerobic mixed cultures, where they obtain energy from the anaerobic oxidation of acetate, growing syntrophically with hydrogen- and formate-utilizing methanogenic archaea [135]. The RC60 sample showed a high percentage of sequences affiliated to the order Dehalococcoidetes (Tab. 6), which comprises obligate organohalide respirers, widely detected in marine and freshwater ecosystems [136], [137]. The presence of organohalide compounds favors the competition with methanogens for the use of molecular hydrogen [138]. Hence the finding of Dehalococcoidetes in the deeper layers of Lake Río Cuarto, retrieved by both pyrosequencing (RC50) and DGGE (RC60), suggest the presence of naturally occurring organo-halogens in the water that could serve as electron acceptors for organohalide-respiring bacteria.

Further confirmation of the importance of anaerobic microbial processes on the CO_2_-CH_4_ balance can be obtained by comparing measured δ^13^C_TDIC_ values with those expected assuming isotopic equilibrium between CO_2_ and HCO_3_^−^. Isotopic fractionation caused by the reaction between dissolved CO_2_ and HCO_3_^−^ is quantified by the enrichment factor (ε_2_), as follows [139]: 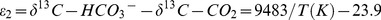
(6)


Theoretical δ^13^C_TDIC_ values (δ^13^C_TDICcalc_) can be computed by:
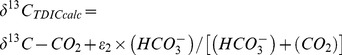
(7)


**Figure 12:**
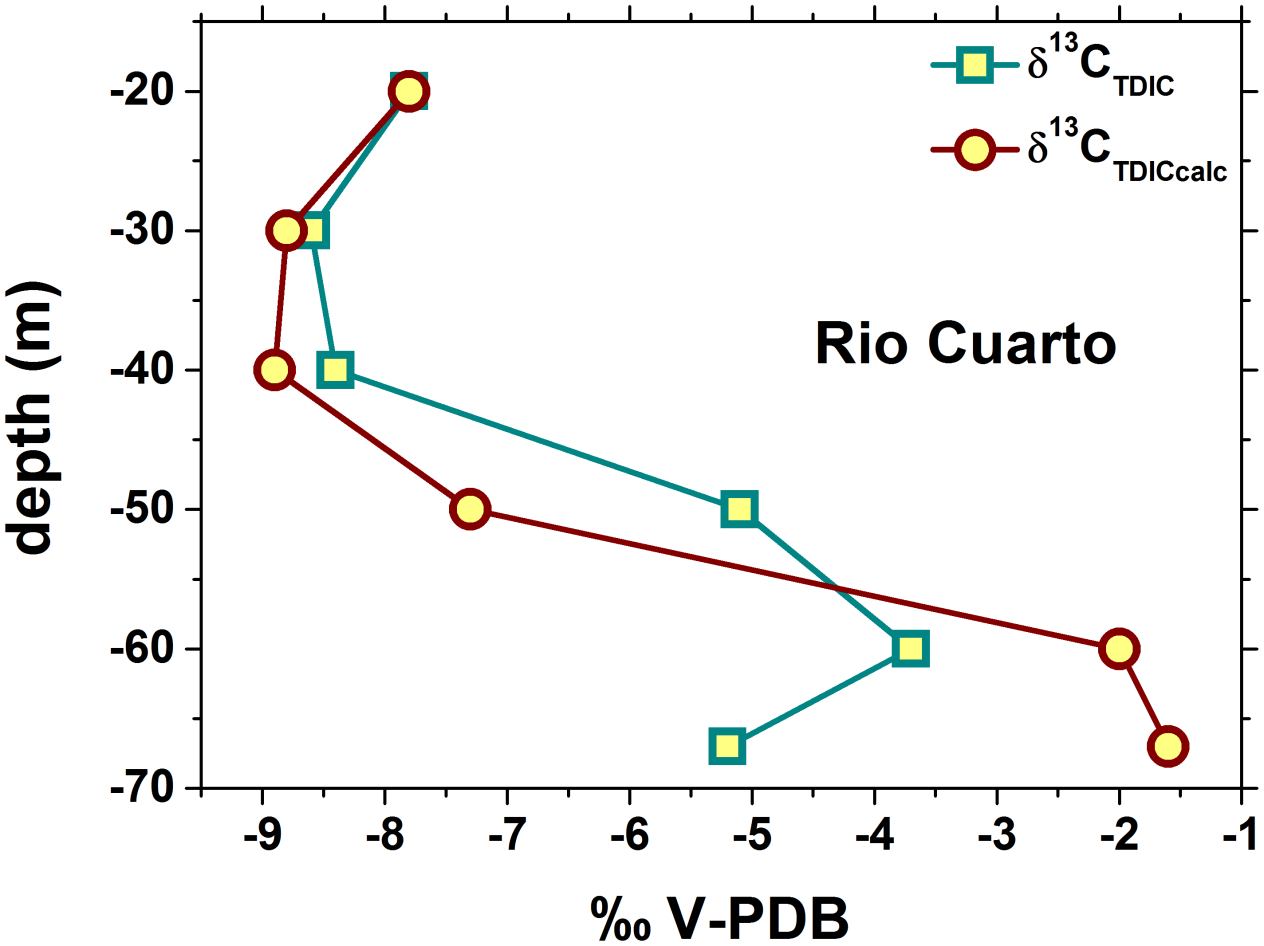
Vertical distribution of measured and calculated δ^13^C_TDIC_ for the water samples from Lake Río Cuarto. See the text for further details.

As shown in Fig. 12, water samples from the shallower strata (down to 40 m depth) of Lake Río Cuarto displayed δ^13^C_TDIC_ and δ^13^C_TDICcalc_ values basically consistent. On the contrary, samples from depth >40 m showed a strong difference between the two sets of values: at −50 m depth, δ^13^C_TDICcalc_ were more negative than δ^13^C_TDIC_, whereas an opposite behavior was observed in the deeper water layer, as well as at the maximum depth of Lake Hule (Tab. 1). At the lake bottoms, continuous inputs of hydrothermal CO_2_, characterized by δ^13^C-CO_2_ values significantly less negative with respect to that already present in the lake, are likely responsible of the positive shift of the δ^13^C_TDICcalc_ values, since this external CO_2_ was not in equilibrium with HCO_3_^−^. In the shallower layers, especially at the depth of −60 m, addition of non-equilibrated biogenic CO_2_ played an opposite role (Fig. 12), whereas at depth ≤40 m CO_2_ concentrations were too low to significantly affect the δ^13^C_TDICcalc_ values, which were consistent with the δ^13^C_TDIC_ ones. The disagreement between measured and calculated δ^13^C_TDIC_ values, depending on both microbial activity and inputs of hydrothermal CO_2_, was documented in other meromictic lakes hosted in volcanic environments, such as Lake Kivu, D.R.C. [34] and the Italian lakes of Albano, Averno and Monticchio [86].

Although the multidisciplinary approach applied in the present study allowed to link the presence of different prokaryotic taxonomic groups to the observed physical conditions and the concentrations of chemical species along the water columns, the ecological function of certain prokaryotes in these intriguing ecosystems, particularly in the case of Lake Hule, remains cryptic. In particular, among the bacterial community, Lake Hule hosted the Actinomycetales ACK-M1 cluster [140], whose phenotypic and metabolic traits have not yet been described. The ACK-M1 cluster was one of the most abundant bacterial taxonomic groups in Lake Hule, reaching up to 22.8% in the oxic water layer H0 (Tab. 6). Moreover, in the Lake Hule waters, the Alphaproteobacterial order Rickettsiales showed relatively high concentrations (18.3% of the total bacterial community in the oxic layer H0; Tab. 6). This order comprises intracellular organisms, pinpointing the importance of symbiotic relationships in these lakes. In this context, the impact of the associations between bacteria and algae [141] or phytoplancton [142] on nutrients re-mineralization was recently discussed showing the crucial role of trophic levels interaction on the food web of lacustrine habitats, possibly relevant also in volcanic lakes.

## Conclusions

Hule and Río Cuarto are meromictic maar lakes mainly fed by meteoric water, and characterized by significant amounts of dissolved gases, partially consisting of CO_2_ having a hydrothermal-magmatic origin, in their hypolimnion. They are currently classified as low activity or, alternatively, “Nyos-type” lakes [4], implying that a limnic eruption could be expected to occur from these lakes, as confirmed by the rollover events they have experienced. However, gases stored in the deep layers of Hule and Río Cuarto are fundamentally different with respect to those of Nyos and Monoun lakes, a difference that must be considered for evaluating the eruption risk. The gas reservoirs of the two Cameroonian killer lakes are composed of almost pure CO_2_ and basically their temporal evolution only depends on a high magmatic gas input rate [12], [13]. At Nyos, the risk of gas bursts was successfully mitigated artificially by discharging the deep-seated gases at the lake surface [35], [143]. On the contrary, the gas reservoirs of Hule and Río Cuarto lakes consist of CO_2_, CH_4_ and N_2_ in comparable amounts, mainly controlled by the activity of a microbial network governed by CO_2_ and CH_4_ metabolism, thus the possible occurrence of a lake rollover that may pose a local risk is not directly related to the input rate of external CO_2_.

Despite geographic separation, Lake Río Cuarto and Lake Hule showed similar physical-chemical settings, though hosting phylogenetically distinct bacterial and archaeal communities. Phylogenetic difference apart, however, both lakes have revealed the presence of the same prokaryotic ecological functions deeply involved in affecting water and gas chemistry.

On the whole, Lake Hule and Lake Rio Cuarto host a CO_2_(CH_4_, N_2_)-rich gas reservoir which is mainly controlled by the complex and delicate interactions occurring between geosphere and biosphere and whose monitoring can appropriately be carried out by coupling the conventional geochemical approach with studies about prokaryotic colonization. Consequently, for these lakes we can introduce the new definition of *bio-activity* lakes. This term can be extended to several other volcanic lakes which show similar compositional features of water and dissolved gases, e.g. Kivu (D.R.C.-Rwanda) [34], [144], Monticchio, Albano and Averno (Italy) [37], [86], [145]–[147], Pavin (France) [121], [148].
